# Phosphorylation induced cochaperone unfolding promotes kinase recruitment and client class-specific Hsp90 phosphorylation

**DOI:** 10.1038/s41467-017-02711-w

**Published:** 2018-01-17

**Authors:** Ashleigh B. Bachman, Dimitra Keramisanou, Wanping Xu, Kristin Beebe, Michael A. Moses, M. V. Vasantha Kumar, Geoffrey Gray, Radwan Ebna Noor, Arjan van der Vaart, Len Neckers, Ioannis Gelis

**Affiliations:** 1 Department of Chemistry, University of South Florida, Tampa, FL 33620 USA; 2 Urologic Oncology Branch, National Cancer Institute, Bethesda, MD 20892 USA

## Abstract

During the Hsp90-mediated chaperoning of protein kinases, the core components of the machinery, Hsp90 and the cochaperone Cdc37, recycle between different phosphorylation states that regulate progression of the chaperone cycle. We show that Cdc37 phosphorylation at Y298 results in partial unfolding of the C-terminal domain and the population of folding intermediates. Unfolding facilitates Hsp90 phosphorylation at Y197 by unmasking a phosphopeptide sequence, which serves as a docking site to recruit non-receptor tyrosine kinases to the chaperone complex via their SH2 domains. In turn, Hsp90 phosphorylation at Y197 specifically regulates its interaction with Cdc37 and thus affects the chaperoning of only protein kinase clients. In summary, we find that by providing client class specificity, Hsp90 cochaperones such as Cdc37 do not merely assist in client recruitment but also shape the post-translational modification landscape of Hsp90 in a client class-specific manner.

## Abstract (web summary)

The Hsp90 chaperone cycle is influenced by multiple phosphorylation events but their regulatory functions are poorly understood. Here, the authors show that phosphorylation and unfolding of cochaperone Cdc37 tailors the Hsp90 chaperone cycle by recruiting kinases that promote distinct phosphorylation patterns.

## Introduction

Heat shock protein 90 (Hsp90) is the core component of a machinery involved in the conformational maturation of a large set of proteins of near-native conformation. It acts upon substrates in the context of a multistep chaperone cycle, which is subject to multiple layers of regulation^1,2^. At the level of the chaperone, the nature of the nucleotide-liganded state controls the population shift between conformations that differ in their local or global molecular architecture^3–6^, as well as in the residence time in each conformation^7^. At the level of the machinery, a large cohort of cochaperones tunes the Hsp90 chaperone cycle^8^. Recruiting cochaperones, such as p60^Hop^ (Sti1) and Cdc37 (p50), act on both the client and the chaperone to stabilize the open Hsp90 conformation, slow down its ATPase activity, and promote efficient client transfer to Hsp90^9–13^. In contrast to Hop, Cdc37 does not function strictly as an adaptor protein. Its selective unfoldase activity on the client over non-client kinases allows for substrate sorting and efficient transfer to Hsp90 by imposing an open kinase conformation^14^ competent for stable chaperone association^15,16^.

As another layer of regulation in eukaryotes, Hsp90 undergoes a plethora of post-translational modifications (PTMs) that include phosphorylation, acetylation, S-nitrosylation, oxidation, SUMOylation, methylation, and ubiquitination^17–19^. Hsp90 PTMs occur as molecular events that assist in the timely progression through the chaperone cycle^20^ or as a response to stimuli such as DNA damage^21,22^ and nitric oxide levels^23^. They have diverse functional consequences that range from altered interaction profiles with clients^24^, cochaperones^20,25^, nucleotides, or small-molecule inhibitors^25^, to translocation^26,27^, secretion^28^, and conformational changes^29–31^. Cochaperones are also subject to PTMs, adding yet another layer of regulation. Phosphorylation of cochaperones modulates their interaction with upstream folding machineries, clients, and Hsp90^16,20,32–34^.

During the kinase chaperone cycle, both Hsp90 and the kinase-specific cochaperone Cdc37 undergo multiple phosphorylation events. The cycle begins with Cdc37 phosphorylated at S13 by CK2, a constitutive modification required for kinase maturation^35,36^. Subsequently, a series of tyrosine phosphorylations on Cdc37 and Hsp90 allow for the disassembly of the substrate-recruitment complex and progression of the cycle^20^. Phosphorylation of Cdc37 at Y4 and Y298 by the non-receptor tyrosine kinase (nRTK) Yes compromises its ability to form complexes with a set of client kinases^20^. Hsp90 phosphorylation at Y197 by Yes or alternative nRTKs results in Cdc37 dissociation and promotes Y313 phosphorylation, which assists in engaging Aha1 into the chaperone complex. The cycle ends with the phosphorylation of Y627, which favors the release of clients and cochaperones^20^. Dephosphorylation is equally important for kinase maturation and the cochaperone phosphatase PP5 was found to act on pS13^34,37^. Finally, Cdc37 phosphorylation at S339 by Ulk1 compromises its ability to associate with protein kinases^38^, while an isoform-specific phosphorylation on Hsp90β by CK2 (S365) compromises its ability to interact with Cdc37^39^.

At a molecular level, the mechanism by which multiple phosphorylation events impact the Hsp90 chaperone cycle remains unexplored. We show that the kinase-specific cochaperone Cdc37 promotes tyrosine phosphorylation of Hsp90 in a client class-specific manner. Our data reveal a mechanism by which specific Hsp90 modification patterns may occur through cochaperone-mediated recruitment of the corresponding modifying enzymes.

## Results

### Y298 hydrogen bonding regulates the conformation of C-Cdc37

**Fig. 1:**
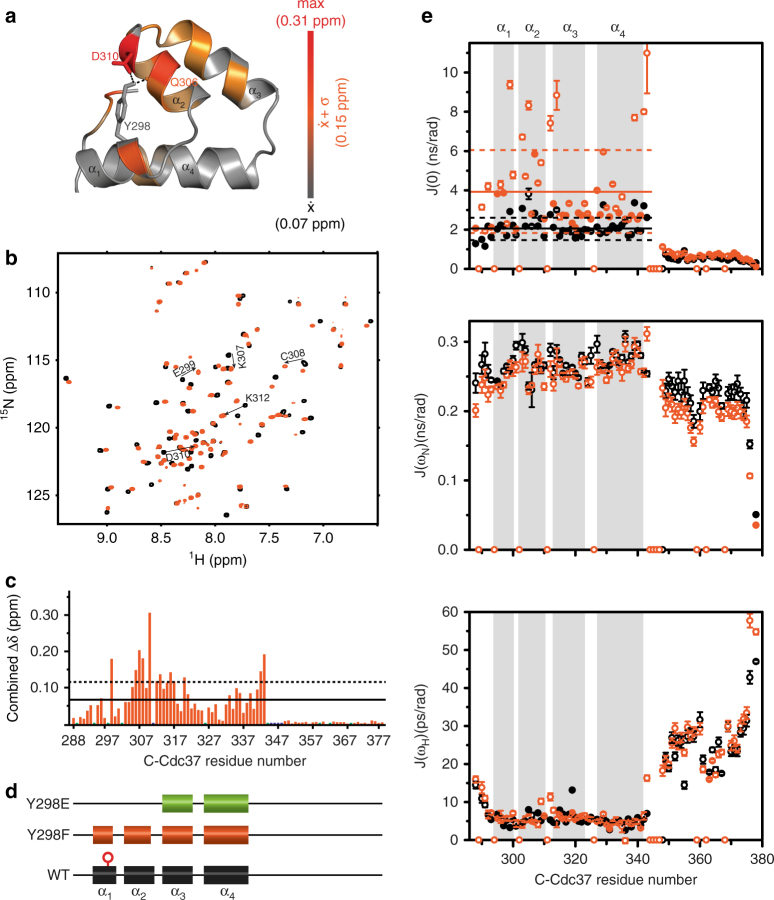
Conformational properties of C-Cdc37^Y298F^. **a** The hydrogen-bond network of Y298 involving Q306 and D310 is shown as black dashed lines on the solution structure of C-Cdc37. C-Cdc37 is colored with a gray-to-red gradient, according to the observed chemical shift perturbation between the wild-type and Y298F C-Cdc37. **b** Overlay of the ^15^N-HSQC of Y298F (orange) and wild-type C-Cdc37 (black) with the tryptophan indole region omitted. **c** Magnitude of CSP between wild-type and C-Cdc37^Y298F^. Prolines are shown as green bars and unassigned residues as blue bars. CSPs higher than the mean or one standard deviation above the mean are marked with solid and dashed lines, respectively. **d** Chemical shift-derived secondary structure for wild-type (black), Y298F (orange), and Y298E (green) C-Cdc37. **e** Reduced spectral density functions *J*(0) (top), J(ω_N_) (middle), and J(0.87ω_H_) (bottom) of Y298F (orange) and wild-type Cdc37 (black). Solid and dashed lines mark the mean and the mean ± one standard deviation of the spectral density functions across the C-Cdc37 sequence

To elucidate the functional role of Y298 phosphorylation in kinase processing, we first investigated the impact of the Y298 hydrogen-bond network on the conformational properties of the C-terminal domain of Cdc37 (C-Cdc37), by introducing the Y298F mutation. In the NMR structure of C-Cdc37^40^, the –OH group of Y298 lies within hydrogen bond distance to the carboxylate group of D310 and the carbonyl group of Q306 (Fig. 1a). We noted that in higher eukaryotes, where phosphorylation regulates activity^20^, Y298 and D310 show a very strong evolutionary covariation, which implies a significant functional coupling between these positions (Supplementary Fig. 1a, b). Comparison of the ^15^N-HSQC spectrum of C-Cdc37^Y298F^ to that of C-Cdc37 reveals that most signals from the folded core of the domain (a.a. 288–343) exhibit significant chemical shift perturbation (CSP), while signals from the flexible C-terminal tail remain unaffected (Fig. 1b, c). The most prominent CSPs are observed for residues in helix α_2_, which encompasses both D310 and Q306, as well as for residues from helix α_3_, which packs against helix α_2_. Notably, irrespective of the magnitude of CSP, all signals appear to shift toward the center of the spectrum, indicating that, as compared to the wild-type domain, there is a relatively small but measurable population shift to an unfolded conformation. This is reflected on both the thermal stability of C-Cdc37^Y298F^, where a 5 °C drop in the T_m_ is observed, and on the intrinsic tryptophan fluorescence, where a 7-nm redshift of the wavelength of maximum emission is observed, indicating a greater exposure (Supplementary Fig. 1c, d). Nevertheless, analysis of the backbone secondary chemical shifts for Cdc37^Y298F^ shows shortening of helices α_1_ and α_2_ by only one and two residues, respectively (Fig. 1d), which is in agreement with the marginal difference in the CD spectrum of the two proteins (Supplementary Fig. 1e).

To further characterize the impact of the Y298 hydrogen bond network on the conformational properties of C-Cdc37, we studied the backbone dynamics of C-Cdc37^Y298F^ by measuring ^15^N relaxation rates (Supplementary Fig. 1f), and adopted the reduced spectral density approach for the analysis^41^ (Fig. 1e). The low-frequency spectral density, *J*(0), is sensitive to both slow (μs–ms) and fast (ps–ns) timescale internal motions. Enhanced μs–ms internal motion is manifested as J(0) values higher than one standard deviation from the mean, while enhanced ps–ns internal motion as J(0) values lower than one standard deviation from the mean. As compared to C-Cdc37, the folded region of C-Cdc37^Y298F^ exhibits higher *J*(0) values, which is reflected by an increase of the mean value from ~2.2 to ~4.0 ns/rad (Fig. 1e). Residues for which significantly large *J*(0) values are observed and thus experience enhanced μs–ms internal motions are E296 and E299 from helix α_1_, E303, L305, C308, and F309 from helix α_2_, K312, V314, and A329 from loop1, and helices α_3_ and α_4_, respectively, as well as residues S339 and W342, at the end of the structured region. Importantly, a similar trend is observed for wild-type C-Cdc37, for which the same set of residues is characterized by large *J*(0) values, albeit of significantly smaller values as compared to C-Cdc37^Y298F^. Hence, although disruption of Y298 hydrogen bond network causes only minimal perturbation in the secondary structure of the folded core, it partially destabilizes its tertiary structure and enhances the μs–ms dynamics at the interface of helices α_1_ and α_2_.

### Y298 phosphorylation results in partial unfolding of C-Cdc37

**Fig. 2:**
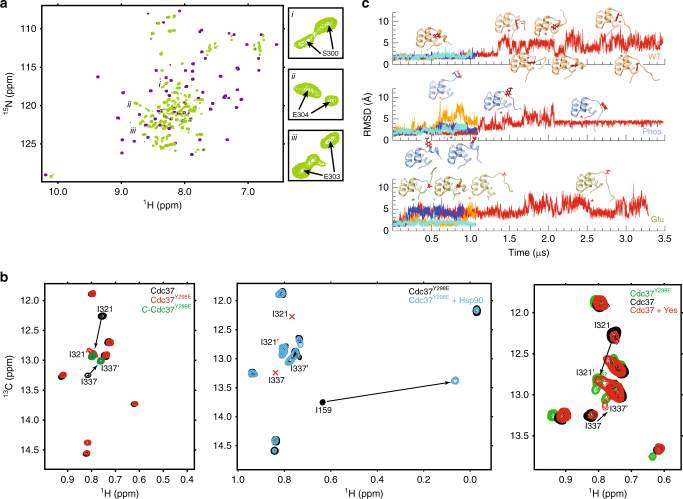
Phosphorylation of C-Cdc37 induces partial unfolding. **a** Overlay of the ^15^N-HSQC of Y298E (green) and wild-type C-Cdc37 (purple) with selected expansions shown on the right. **b** Selected regions (Ile^δ^) from ^13^C-HMQC spectra of Cdc37 constructs. Left: overlay of full-length wild-type Cdc37 (black), full-length phosphomimetic mutant Cdc37^Y298E^ (red), and C-domain phosphomimetic mutant C-Cdc37^Y298E^ (green). Middle: overlay of free Cdc37^Y298E^ (black) and in the presence of one equivalent deuterated Hsp90α (cyan). The red marks indicate the positions of I321 and I337 signals in wild-type Cdc37. Right: overlay of Cdc37 (black) and Cdc37 in the presence of 15-μg Yes acquired in “phosphorylation” buffer (red), together with Cdc37^Y298E^ (green). **c** Backbone rmsd for unmodified (top), phosphorylated (middle), and C-Cdc37^Y298E^ (bottom). Simulations at 300 K are shown in dark and light blue, and at 310 K in orange and red. Stars mark the time intervals from which snapshots were extracted

We next sought to investigate the effect of phosphoryl group addition to the side chain of Y298 on the conformational properties of C-Cdc37, by introducing the phosphomimetic mutation Y298E. In contrast to the ^15^N-HSQC spectrum of C-Cdc37^Y298F^, which shows only small changes in signal dispersion and linewidths as compared to C-Cdc37, the fingerprint spectrum of C-Cdc37^Y298E^ exhibits poor signal dispersion suggesting that the introduction of a negative charge on the side chain of Y298 has a global effect on the folded region of C-Cdc37 and brings a significant loss of the native structure (Fig. 2a). This observation is further supported by a 13-nm redshift in the wavelength of maximum tryptophan emission, to a position between the emission maxima of C-Cdc37^Y298F^ and that of C-Cdc37 acquired in the presence of 9 M urea (Supplementary Fig. 1d). Still, as evident by the far-UV CD spectra, C-Cdc37^Y298E^ retains some secondary structure (Supplementary Fig. 1e), while its ^15^N-HSQC spectrum displays very broad linewidths. Hence, loss of the native structure by the phosphomimetic mutation does not result in domain disorder but rather in the transition of C-Cdc37 to a conformationally heterogeneous, partially unfolded state. This effect is not an artefact caused by domain truncation. Comparison of the ^13^C-HMQC or ^15^N-HSQC spectra of full-length Cdc37 carrying the phosphomimetic mutation (Cdc37^Y298E^) to that of the wild-type protein and of C-Cdc37^Y298E^ (Fig. 2b and Supplementary Fig. 2a) reveals a global loss of dispersion for the signals of the C domain similar to that observed for the isolated C-Cdc37^Y298E^. The new signals that appear in the “unfolded” region of the spectrum of full-length Cdc37^Y298E^ show very good chemical shift correspondence to those of isolated C-Cdc37^Y298E^, supporting a shift to the same partially unfolded state. In addition, partial unfolding is not affected by the presence of Hsp90. Although Cdc37^Y298E^ forms a stable complex with Hsp90 as reported by the large chemical shift change of I159, which lies at the vicinity of the Cdc37-Hsp90 interface, the C-domain signals of I321 and I337 remain unperturbed at the unfolded region (Fig. 2b).

In order to assess whether partial unfolding due to the phosphomimetic mutation is genuine and recapitulates the effect caused by the addition of a phospholyl group at position Y298, we first monitored the Yes-mediated phosphorylation of Cdc37 by NMR. Although the reaction was ~40% complete, the new signals that appear in the ^13^C-HMQC spectrum of Cdc37 overlay well with the CH_3_^δ^ signals of I321 and I337 of Cdc37^Y298E^, suggesting that the tyrosine-to-glutamate substitution behaves as a faithful phosphomimetic in shifting the conformation of the C domain to a partially unfolded state (Fig. 2b). We also compared the molecular dynamics simulations of wild-type, Y298E, and pY298 C-Cdc37, performed at two temperatures (Fig. 2c and Supplementary Fig. 2b). At 300K, C-Cdc37 remained folded in both simulations, and native contacts between helices α_1_ and α_2_ were maintained. On the other hand, for pY298, near the end of the simulations, helix α_1_ rotated toward the solvent, resulting in loss of native contacts with helix α_2_, while in one of the Y298E simulations, α_1_ unfolded and native contacts with α_2_ were lost. At 310 K, helix α_1_ unfolded and contacts between residue 298 and helix α_2_ were lost, for both pY298 and Y298E C-Cdc37, while for C-Cdc37, unfolding of helix α_1_ was only observed in the extended simulation, but without loss of native contacts and with several short refolding events. In summary, the disruption of the hydrogen-bonding network of Y298 and the addition of a negatively charged group have a synergistic effect on the conformational properties of C-Cdc37, resulting in loss of native secondary and tertiary structure.

### C-Cdc37^pY298^ populates native-like folding intermediates

**Fig. 3:**
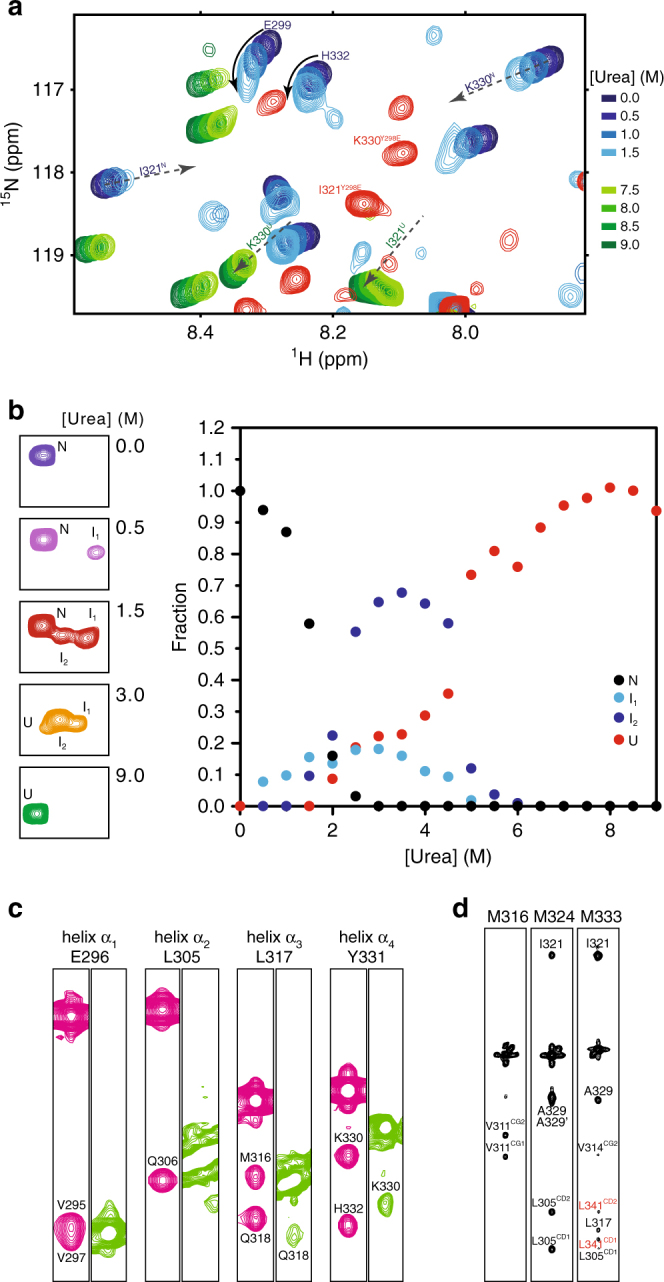
Phosphorylated C-Cdc37 populates folding intermediates. **a** The beginning (0.0–1.5 M urea) and end (7.5–9.0 M urea) of the equilibrium unfolding of C-Cdc37 monitored by ^15^N-HSQC. Arrows track the shift of signals from the native to the unfolded state. For I321 and K330 for which the signals of both the native and unfolded states are visible in this expansion, the superscripts N and U on the assignment denote native and unfolded states, respectively. For E299 and H332, the signals of the unfolded state fall outside the present spectral window, but show a “curved” change in chemical shift with increasing urea concentration. The spectrum of C-Cdc37^Y298E^ is shown in red for comparison highlighting the position of I321 and K330 signals. **b** The indole H–N region of wild-type C-Cdc37, highlighting the signals that correspond to W342 at different points of the equilibrium unfolding (left). Fractional populations of the four visible species populated by C-Cdc37 during the equilibrium unfolding as a function of urea concentration (right). **c** Selected strips from the amide region of the ^15^N-NOESY-HSQC recorded for the wild-type C-Cdc37 (pink) and C-Cdc37^Y298E^ (green). Representative residues from all helices α_1_–α_4_ are included. **d** Selected strips from the HMQC–NOESY–HMQC spectrum of C-Cdc37^Y298E^, highlighting unambiguous (black) and tentative (red) NOEs

To understand the nature of the unfolding transition triggered by the phosphorylation of Y298, we examined the unfolding of C-Cdc37 under equilibrium conditions. The ^15^N-HSQC spectra of C-Cdc37 acquired in the presence of increasing concentrations of urea show that, for most residues of the structured core, unfolding occurs on the intermediate fast-exchange regime (Supplementary Fig. 3a). From those resonances that are unambiguously traced at both low- and high-urea concentrations, it is evident that the observed shift toward the unfolded state does not follow a linear, but either an angular or a curved trajectory (Fig. 3a). Therefore, the unfolding transition cannot be described based on a simple two-state model where the native state (N) is in equilibrium with the unfolded state (U), but intermediate species must be considered. Observing the indole resonance of W342, which is well resolved and the only resonance that shifts on a slow-exchange fashion without any apparent line broadening, reveals that during the course of the titration at least two folding intermediate states, I_1_ and I_2_, become highly populated between 0.5 M and 5.5 M urea (Fig. 3b). I_1_ appears at the beginning of the unfolding transition (0.5 M urea) and reaches a maximum fractional population of ~0.2, followed by I_2_ (1.5 M urea), which reaches a maximum fractional population of ~0.7. Notably, during the early stage of unfolding (~2.0 M urea), the sum of fractional populations is significantly lower than 1.0, indicating that a fraction of C-Cdc37 (~0.4) populates alternate conformational states that are invisible under the current experimental conditions, presumably because they interconvert on a millisecond timescale. Comparison of the C-Cdc37 spectra acquired during the unfolding transition to that of C-Cdc37^Y298F^ and C-Cdc37^Y298E^ provides further insights as to how the composition of the conformational ensemble sampled by C-Cdc37 is altered in response to the properties of the side chain at position 298 (Supplementary Fig. 3b and 3c). Most resonances in the ^15^N-HSQC spectrum of C-Cdc37^Y298F^ overlay well with resonances of C-Cdc37’s spectrum acquired in the presence of 1.5 M urea. Hence, disruption of Y298 hydrogen-bonding network results in a population shift within the conformational ensemble to a state where I_1_ becomes populated. On the other hand, most resonances of C-Cdc37^Y298E^ overlay well with resonances of C-Cdc37’s spectrum acquired in the presence of 4.5 M urea, suggesting that the introduction of a negative charge at position 298 produces a partially unfolded state, where folding intermediate I_2_ is significantly populated. Backbone assignment of C-Cdc37^Y298E^ reveals that in the phosphorylated state, only helices α_3_ and α_4_ are fully formed, helix α_2_ shows a low propensity of formation at very low confidence, and the sequence covered by helix α_1_ adopts a coil conformation (Fig. 1d and Supplementary Fig. 3d). This is further supported by the presence of dNN(*i*,*i* + 1) NOEs for residues from helices α_3_ and α_4_, which are characteristic of α-helical structure, and the absence of NOEs for residues forming helices α_1_ and α_2_ in the native state (Fig. 3c). In addition, for a set of eight residues (E298, E299, S300, E303, E304, I321, L341, and V343), it was possible to unambiguously assign two signals, both of which exhibit an irregular lineshape, indicating a higher degree of heterogeneity than what is reported by the W342^ε^ signal (Fig. 2a). This set of residues is not clustered, but it is distributed throughout the sequence and structure of the native state, as expected based on the global changes observed in the spectrum. To obtain further insights into the structural properties of the partially unfolded state, we acquired the methyl-NOESY of C-Cdc37^Y298E^. The observed CH_3_-CH_3_ NOE pattern includes a small number of medium- and long-range NOEs from helices α_3_, α_4_, and residues comprising helix α_2_, indicating that the phosphomimetic mutant samples a conformational ensemble that is stabilized by native-like contacts between helices α_2_–α_4_ (Fig. 3d).

Thus, phosphorylation of Cdc37 by Yes results in partial unfolding of C-Cdc37 and shift to a heterogeneous conformational ensemble which comprises at least two equilibrium-folding intermediates of near-native conformation.

### Coupled Cdc37^Y298^ and Hsp90^Y197^ tyrosine phosphorylation

**Fig. 4:**
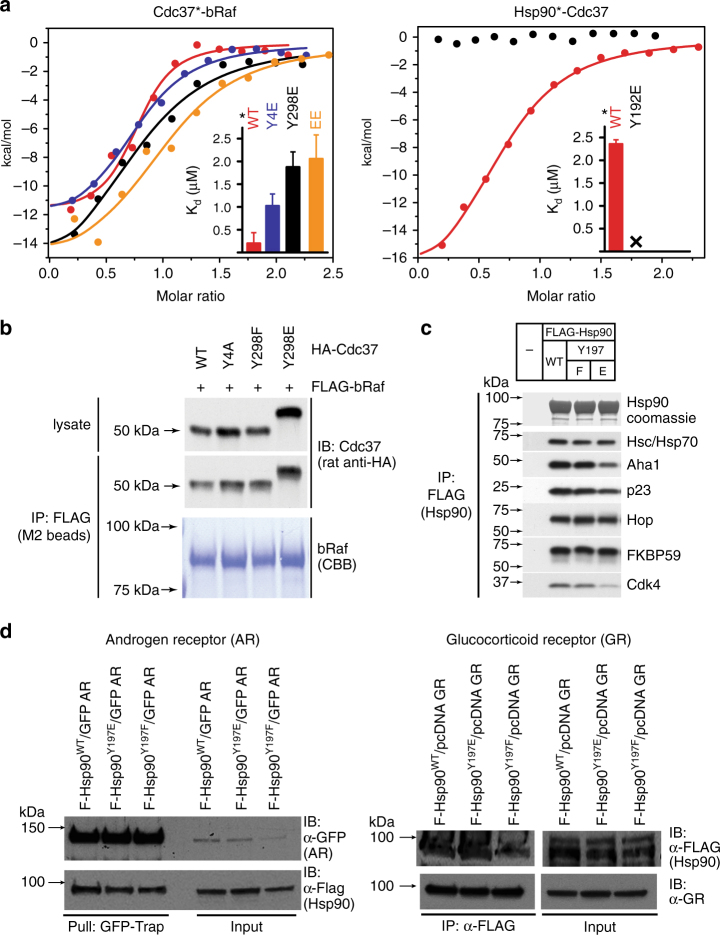
Assembly of complexes of Cdc37 and Hsp90 phosphomimetic variants with clients and cochaperones. **a** Binary complex formation between Cdc37 variants and bRaf (left), and between Hsp90β variants and Cdc37 (right), followed by ITC. The corresponding *K*_d_ values are displayed in the inset. Error bars in the *K*_d_ values correspond to the errors resulted in fitting of the data into a single binding site model. **b** HEK-293 cells were cotransfected with indicated HA-tagged Cdc37 and FLAG-tagged bRaf plasmids. After cell lysis, proteins were immunoprecipitated with anti-FLAG resin for 1 h at 4 °C with rotation. Bead pellets were washed and analyzed for Cdc37 interaction by SDS-PAGE/western blot, using anti-HA antibody. **c** HEK-293 cells were transfected with FLAG-tagged Hsp90, Hsp90^Y197E^, or Hsp90^Y197F^ plasmids. After cell lysis, proteins were immunoprecipitated with anti-FLAG resin for 1 h at 4 °C with rotation. Bead pellets were washed three times before analysis by SDS-PAGE/western blot. Co-precipitating endogenous Hsp70, Aha1, p23, Hop, Fkbp59, and Cdk4 were detected with specific antibodies. **d** HEK-293 cells were transfected with the indicated Hsp90, androgen receptor (AR), and glucocorticoid receptor (GR) plasmids. Proteins were precipitated with GFP-Trap resin (left) or ANTI-FLAG M2 agarose (right) for 1 h at 4 °C with rotation. Bead pellets were washed three times with lysis buffer before analysis by SDS-PAGE/western blot as indicated. AR was visualized with anti-GFP antibody, GR was visualized with a specific antibody, and Hsp90 was visualized with anti-FLAG antibody

Next, we investigated the molecular mechanism by which partial unfolding of C-Cdc37 regulates the Hsp90 chaperone cycle of protein kinases. Y298 phosphorylation has been implicated in the dissociation of ErbB2, Cdk4, Raf-1, and v-Src from Cdc37, without affecting the interaction of Cdc37 with Hsp90^20^. Since C-Cdc37 is directly involved in the formation of binary Cdc37-kinase complexes^14,42^, we used purified proteins to monitor the formation of binary and ternary complexes of Cdc37 Y298 variants with bRaf and Hsp90. We found that the phosphomimetic mutant Y298E forms stable binary complexes with both bRaf and Hsp90, and ternary complexes with bRaf and Hsp90 that are indistinguishable from those formed by the wild-type protein^14,43^ (Fig. 4a and Supplementary Fig. 4a, b). Despite the higher *K*_d_ value observed for the interaction between Cdc37^Y298E^ and bRaf as compared to wild- type Cdc37, this observation is distinct from previously reported findings using co-immunoprecipitation from cell lysates^20^. Therefore, we also tested complex formation with the single phosphomimetic mutant Y4E (Cdc37^Y4E^) or the double-phosphomimetic mutant Y4E/Y298E (Cdc37^EE^). Phosphorylation of Y4, which is located at the N-terminal tail of Cdc37, was previously shown to have a more limited impact, affecting the association of Cdc37 only with ErbB2 and v-Src. As for Cdc37^Y298E^, both Cdc37^Y4E^ and Cdc37^EE^ formed stable binary complexes with bRaf and Hsp90, and ternary complexes with bRaf and Hsp90 (Fig. 4a and Supplementary Fig. 4a, b). Next, we tested complex formation in an in vivo setting, where Flag-tagged bRaf was immunoprecipitated from transiently transfected HEK-293 cells and the associated HA-tagged Cdc37 variants were visualized by western blotting. Similarly to the in vitro experiments, both phosphomimetic Cdc37^Y298E^ and the non-phosphomimetic mutant Cdc37^Y298F^ were identified in bRaf complexes to the same extent as was wild-type Cdc37 (Fig. 4b and Supplementary Fig. 5).

In addition to Cdc37 phosphorylation at positions Y4 and Y298, Yes and other nRTKs phosphorylate Hsp90 at positions Y197 and Y192 of the α and β isoforms, respectively. This modification correlates with disassembly of Cdc37 from the substrate-recruitment complex and promotes progression of the chaperone cycle^20^. In agreement with previous data^20^, Hsp90^Y192E^ did not form stable binary complexes with Cdc37 and/or ternary complexes with Cdc37 and bRaf (Fig. 4a and Supplementary Fig. 4a, b). Therefore, Cdc37 phosphorylation at Y298 does not have a generalized impact on the formation of binary or ternary complexes with Hsp90 and the client kinase, but instead, it is the phosphorylation of Hsp90 at Y192/Y197 which acts as the molecular switch that triggers disassembly of the substrate-recruitment complex and cochaperone dissociation. Importantly, modification of this residue has minimal impact on the association of Hsp90 with other cochaperones, including that of Hsp70, Aha1, p23, Hop, Fkbp59, Sugt1, and CHIP or with non-kinase clients, including the androgen and glucocorticoid receptors (Fig. 4c, d and Supplementary Figs. 4c, d and 5).

**Fig. 5:**
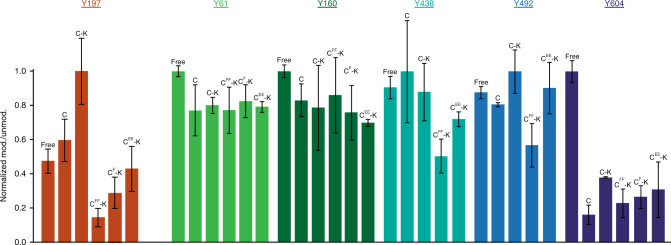
Phosphorylation of Hsp90 in the context of in vitro-assembled complexes. Mass spectrometry intensity ratios of Yes-modified Hsp90α peptides over the corresponding unmodified peptides for positions Y61, Y160, Y197, Y438, Y492, and Y604. Ratios are normalized to the highest-detected phosphorylation levels among five different liganded states of Hsp90α: free, in a binary complex with wild-type Cdc37 (C), or in a ternary complex with bRaf and Cdc37 variants (wild type = C-K, doubly non-phosphorylatable = C^FF^-K, singly non-phosphorylatable = C^F^-K, and double phosphomimetic = C^EE^-K). Error bars are defined as s.d., over two replicates

Since both Cdc37^Y298^ and Hsp90^Y197^ phosphorylation are mediated by Yes, we investigated whether the functional consequence of Cdc37^Y298^ phosphorylation and C-Cdc37 partial unfolding is to regulate Hsp90^Y197^ phosphorylation. Purified variants of Cdc37 and Hsp90 were utilized to assemble binary and ternary complexes formed with the client bRaf, and their tyrosine phosphorylation levels in the presence of added Yes were quantified using mass spectrometry (Fig. 5 and Supplementary Fig. 6). The highest level of Yes-mediated Cdc37^Y298^ phosphorylation was detected in the free state or when in a binary complex with bRaf, while reduced phosphorylation was observed for both a ternary complex between Cdc37, Hsp90, and bRaf, and for a binary complex of Cdc37 with Hsp90 (Supplementary Fig. 6a). However, the total cellular pool of Cdc37 remains in a hypophosphorylated state, as phosphorylation at either Y4 or Y298 is only detected after treatment with the potent phosphotyrosine phosphatase inhibitor bpv(phen)^20^. Therefore, the significant difference in the phosphorylation levels between the free and complexed states of Cdc37 observed in the current in vitro setting is suppressed in the cell, presumably due to the action of protein phosphatases. On the other hand, Hsp90^Y197^ phosphorylation was markedly more efficient in the context of a ternary complex with Cdc37 and bRaf, compared to either the free state or in a binary complex with Cdc37 (Fig. 5). Importantly, such dependence on a ternary complex for optimal phosphorylation was not observed for other Hsp90 tyrosines that were detectably phosphorylated in the presence of Yes. Similarly, Cdc37^Y298^ mutation did not have a marked impact on Yes-mediated phosphorylation of these additional sites (Fig. 5). However, when the non-phosphorylatable Y4F/Y298F double mutant (Cdc37^FF^) or the single non-phosphorylatable mutant Y298F (Cdc37^F^) were utilized to reconstitute ternary complexes, Hsp90^Y197^ phosphorylation dropped by one order of magnitude (Fig. 5). Intriguingly, in ternary complexes of the double-phosphomimetic mutant Cdc37^EE^, Hsp90^Y197^ phosphorylation was also compromised and still lower relative to ternary complexes of the wild-type protein (Fig. 5). These results suggest that Yes-mediated Cdc37^Y298^ phosphorylation uniquely promotes Hsp90^Y197^ phosphorylation and that the Hsp90^Y197^-dependent assembly and disassembly of the recruitment complex are characterized by a coupled phosphorylation mechanism. Therefore, coupled Hsp90^Y197^ phosphorylation does not merely depend on the partial unfolding of C-Cdc37, but specifically requires that the amino acid at position 298 of Cdc37 is a phosphorytable tyrosine.

### C-Cdc37 unfolding unmasks a high-affinity SH2-binding motif

**Fig. 6:**
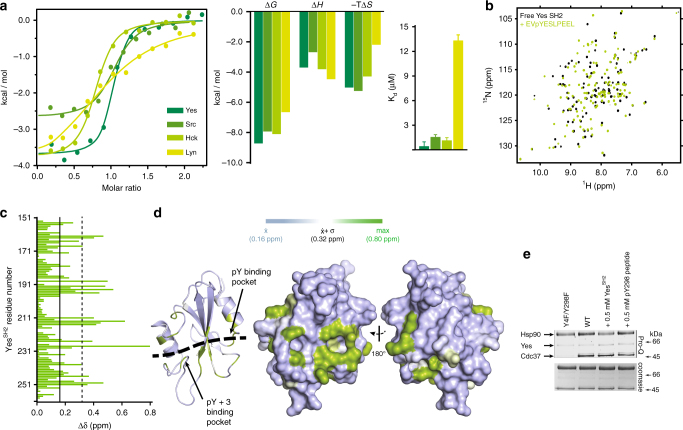
Interaction of a pY298 phosphopeptide with the SH2 domains of Yes and other nRTKs. **a** ITC isotherms for the interaction of pYESL with the SH2 domains of Yes, Src, Hck, and Lyn (left), together with the enthalpic and entropic contributions to the free energy change (middle) and the corresponding *K*_d_ values (right). Error bars in the *K*_d_ values correspond to the errors resulted in fitting of the data into a single binding site model. **b** Overlay of the ^15^N-HSQC of Yes^SH2^ in the absence (black) and presence (green) of one equivalent of pYESL. **c** CSP as a function of Yes^SH2^ primary sequence. The mean and one standard deviation above the mean are marked by solid and broken lines, respectively. **d** Mapping of the observed CSPs on the structure of SH2. The black broken line highlights the peptide-binding site as identified in other SH2 domains. **e** The effect of competing concentrations of SH2^Yes^ or pYESL (at 0.5 mM) on the overall phosphorylation levels of Hsp90 in the context of ternary complexes formed with bRaf and Y4F/Y298F (lane 1) or wild-type Cdc37 (lanes 2–4), monitored by staining with Pro-Q Diamond (top) and coomassie (bottom)

The dependence on Cdc37 in uniquely facilitating Hsp90^Y197^ phosphorylation on a phosphotyrosine indicates that coupling of the two phosphorylation events is characterized by high specificity for pY298. The vast majority of nRTKs contain N-terminal SH2 and SH3 modular domains, among which the former mediates protein–protein recognition through a specific interaction with short polypeptide stretches that contain a phosphotyrosine. Thus, we tested the hypothesis that promotion of Hsp90^Y197^ phosphorylation by Cdc37 phosphorylated at Y298 occurs through an SH2-mediated recruitment of Yes to Hsp90. Scanning of the primary sequence of Cdc37 for conserved motifs using highly stringent parameters^44,45^ identified the heptapeptide ^296^EVYESLP^302^, which encompasses phosphotyrosine Y298, as an SH2 interaction motif, while using low-stringency parameters the tetrapeptide ^359^PGDP^362^ is identified as a potential SH3 interaction motif. When a phosphotyrosine-modified decapeptide of Cdc37 (denoted as pYESL) was tested for binding to the SH2 domain of Yes (Yes^SH2^), it was indeed found to exhibit high affinity (*K*_d_ = 0.41 ± 0.11 μM) (Fig. 6a). The interaction is driven by thermodynamically favorable contributions of both the enthalpic and the entropic terms, which is similar to the thermodynamic signature of binding for the interaction of phosphopeptides to Src SH2 domain (Src^SH2^)^46^ (Fig. 6a). Several structural studies of SH2 domains in complex with phosphopeptides have revealed a conserved mode of recognition, where the phosphotyrosine and the three residues immediately downstream are recognized by distinct binding pockets. Although to date the mode of Yes^SH2^-phosphopeptide interaction has not been studied at a structural level, we used NMR to examine whether the Yes^SH2^-pYESL interaction maps on a surface common to other SH2-phosphopeptide complexes. Titration of pYESL to ^15^N-labeled Yes^SH2^, results in CSP of a large number of signals in an intermediate-slow exchange fashion, consistent with the affinity measured by ITC (Fig. 6b, c and Supplementary Fig. 7a). Mapping the CSP on the structure of Yes^SH2^ shows that the most prominent changes are observed for residues in the vicinity of the putative pTyr and pTyr + 3 binding pockets, showing excellent correspondence with the residues involved in phosphopeptide recognition of other SH2 domains (Fig. 6d and Supplementary Fig. 7b). Furthermore, the addition of C-Cdc37^Y298E^ in which helix α_1_ is unfolded or wild-type C-Cdc37 in which helix a1 is fully formed cause only minimal perturbation in the ^15^N-HSQC of Yes^SH2^ (Supplementary Fig. 7c). Therefore, Cdc37 phosphorylated at position Y298 is recognized by Yes^SH2^ through a typical SH2-phoshopeptide interaction.

A potential mechanism by which Yes recruitment through the SH2-Cdc37^pY298^ recognition affects Hsp90 phosphorylation is by increasing the local concentration of the modifying enzyme at a particular location on Hsp90. To test this hypothesis, we assessed the total Hsp90 phosphorylation in the context of ternary complexes and in presence or absence of an excess of SH2^Yes^ or pYESL, using the Pro-Q Diamond stain to detect phosphotyrosines (Fig. 6e). Although addition of pYESL had a moderate impact on the overall levels of Hsp90 phosphorylation (7% drop), addition of SH2^Yes^ resulted in a substantial 25% drop, consistent with the concept that an excess of the isolated SH2 domain is able to compete with full-length Yes for binding to Cdc37^pY298^ and exclude it from the chaperone complex, even though Yes-mediated phosphorylation of other Hsp90 tyrosine residues appear to be independent of Cdc37.

Phosphorylation of Hsp90 at position Y197, as well as at positions Y313 and Y627 is not carried out solely by Yes, since Yes knockdown fails to completely abolish phosphorylation of these residues^20^. This indicates that other nRTKs can substitute for Yes in recognizing Cdc37^pY298^ and may be recruited to the Hsp90 complex through a SH2-phoshopeptide interaction. Thus, we examined the extent to which pYESL can be recognized by the SH2 domains of nRTKs of the Src kinase family other than Yes, including those of Src, Hck, and Lyn. All domains show similar thermodynamic signatures of binding, with the Src^SH2^ and Hck^SH2^ having *K*_d_ values comparable to those of Yes^SH2^, while as expected based on the *K*_d_, addition of pYESL into ^15^N-labeled Src^SH2^ results in large CSPs to its ^15^N-HSQC (Fig. 6a and Supplementary Fig. 7d). On the other hand, the interaction with Lyn^SH2^ is significantly weaker, indicating that not all SH2 domains can effectively recognize pYESL and thus substitute for Yes. Finally, we tested whether the ^359^PGDP^362^ motif of C-Cdc37 is recognized by the SH3 domain of Yes (SH3^Yes^) and therefore whether it provides additional specificity determinants and stability to the Cdc37-Yes complex. Addition of excess Cdc37 to ^15^N-labeled SH3^Yes^ only marginally affects its ^15^N-HSQC spectrum, with very small chemical shift changes and broadening for a small number of signals, indicating a weak, transient interaction, which is expected considering the lack of flanking proline or positively charged residues. Nevertheless, perturbed residues map at the canonical SH3-binding site and include R105 and T106 of the conserved RT loop of SH3 domains (Supplementary Fig. 7e).

In summary, the functional role of tyrosine phosphorylation-induced partial unfolding of C-Cdc37 is to unmask a high- affinity SH2-binding motif that serves to increase the local concentration of nRTKs at Hsp90 and thus to potentiate Hsp90 tyrosine phosphorylation at specific sites.

## Discussion

**Fig. 7:**
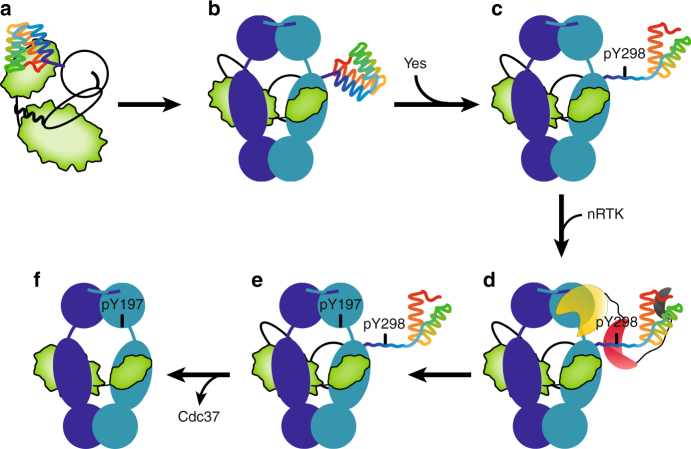
Targeted-Hsp90 phosphorylation via a cochaperone-recruited kinase. **a**, **b** Client kinases require Cdc37 to form stable complexes with Hsp90 in the beginning of the chaperone cycle. **c**, **d** Partial unfolding of C-Cdc37 upon Y298^Cdc37^ phosphorylation creates a high-affinity SH2-interacting motif, which recruits nRTKs to the Hsp90 complex. **e**, **f** nRTKs phosphorylate Hsp90 at Y197, resulting in dissociation of Cdc37 and progression of the Hsp90 cycle. Cochaperone-mediated recruitment of modifying enzymes may be a generalized mechanism to create unique Hsp90 phosphorylation patterns in a client class-specific manner or alternatively to modify clients (Hsp90, Cdc37, and client kinase are shown in blue, black outline, and green, respectively). The helical C-Cdc37 is shown in rainbow from blue to red. The kinase, SH2, and SH3 domains of the modifying nRTK are shown in yellow, red, and gray, respectively

Phosphorylation of Hsp90 and its composite machinery exerts key regulatory roles during client maturation. Here, we show that during the kinase chaperone cycle, Cdc37 phosphorylated at Y298 acts as a platform for docking of non-receptor tyrosine kinases through their regulatory domains to drive the coupled Hsp90 phosphorylation at Y197 and specifically regulate kinase chaperoning (Fig. 7).

Site-specific phosphorylation of Hsp90 has a differential impact on the maturation of different classes of clients, including protein kinases and transcription factors^25,27,47^. Our study suggests that the cochaperone-mediated recruitment of modifying enzymes provides a mechanism to generate highly specific Hsp90 modification patterns (Fig. 5) that are tailored to fine-tune the chaperone cycle in a client class-specific manner. We show that Hsp90 phosphorylation at Y197 does not compromise association of the Cdc37-independent nuclear hormone receptor clients glucocorticoid receptor (GR) and androgen receptor (AR) with the chaperone (Fig. 4d), while a clear reduction in Cdk4 interaction with the phosphomimetic mutant Hsp90-Y197E is evident (Fig. 4c). Further, Hsp90^Y197E^ fails to associate with Cdc37 (Fig. 4a and Supplementary Fig. 4b), which during the early stages of the chaperone cycle interacts exclusively with the N-terminal domain of Hsp90^11,15,16,48^, without losing its ability to associate with a number of cochaperones (Fig. 4c and Supplementary Fig. 4c, d) that interact either with the C-terminal domain or with the middle and N-terminal domains of Hsp90.

Phosphorylation of the Hsp90-Cdc37 chaperone pair has been largely explored; however, there is only limited high-resolution information available to account for the functional outcome of the observed modifications^14,16^. The addition of a phosphoryl group may alter protein function through versatile mechanisms that include allosteric changes^49^, direct positive or negative modulation of protein–protein and protein–DNA interactions^50^, availability of cofactor-binding sites^51^, autoinhibition^52^, disorder-to-order^53^, and order-to-disorder^54^ transitions. Our NMR data suggest that C-Cdc37 acquires a partially unfolded state when phosphorylated at Y298 (Fig. 2 and Supplementary Fig. 2). This is a synergistic effect caused by the disruption of the hydrogen bond network of Y298 side chain and the addition of the phosphoryl group (Figs. 1, 2). The folding-unfolding transition of a small protein domain of the size of C-Cdc37 occurs typically in a two-state manner, where only the native and unfolded states are populated. However, folding of C-Cdc37 through a highly populated folding intermediate (Fig. 3 and Supplementary Fig. 3) provides the cochaperone with two unique functional advantages. First, helices α3 and α4 that form a hydrophobic patch previously shown to participate in binding bRaf^14^, are fully formed (Figs. 1,3 and Supplementary Fig. 3d), allowing Cdc37 to interact with clients and Hsp90 in the context of binary and/or ternary complexes (Fig. 4 and Supplementary Fig. 4). Second, helix α1, which contains Y298, is unfolded in the phosphorylated state (Figs. 2,3). This phosphorylation-stabilized extended conformation unmasks a high-affinity SH2-binding phosphopeptide, which exhibits broad specificity over SH2 domains of nRTKs (Fig. 6). These include the SH2 domain of the Cdc37- modifying enzyme Yes, as well as the SH2 domains of other nRTKs of the Src family that are capable of phosphorylating Hsp90^Y197^^20^.

A common concern in using phosphomimetic mutants, particularly for tyrosine and less for serine and threonine phosphorylation, is whether these serve as bona fide mimics of the authentic phosphorylated state. Our results suggest that for Cdc37, Y298 phosphorylation facilitates two distinct molecular events and functional outcomes. First, partial unfolding of C-Cdc37, which results in the formation of an exposed phosphopeptide sequence, and second, recognition of the resulting phosphopeptide by SH2 domains of nRTKs, which potentiates Hsp90^Y197^ phosphorylation. We show that the partially unfolded state acquired by Cdc37^Y298E^ is the same as the one populated after Yes-mediated phosphorylation (Fig. 2b). Therefore, the phosphomimetic mutant is a faithful mimic in initiating the native conformational change produced by the phosphorylation event. However, the “two-pronged plug into two-holed socket” mode of SH2-phosphopeptide interactions imposes specific geometric and chemical restraints for the binding pockets and particularly for the pY-binding pocket, which is optimized for interacting with the phosphoryl group^55,56^. Evidently, the corresponding phosphomimetic sequence in C-Cdc37^Y298E^ fails to form a stable complex with SH2^Yes^ (Supplementary Fig. 7c). Therefore, in this case, the Y298E phosphomimetic mutant is not a faithful mimic in recruiting non-receptor tyrosine kinases to the chaperone complex via SH2-mediated interactions, consistent with our data showing reduced Hsp90^Y197^ phosphorylation levels for complexes reconstituted with the non-phosphorylatable mutants as compared to wild-type Cdc37-reconstituted complexes (Fig. 5).

The binding of the Cdc37-derived phosphopeptide to SH2 domains is not the only non-kinase domain interaction identified here, as C-Cdc37 interacts transiently also with the SH3 regulatory domain of Yes (Supplementary Fig. 7e). Although weak, it is expected that this interaction brings a significant stabilizing contribution when coupled to the C-Cdc37^pY298^-SH2 interaction in the context of a bipartite mode of binding. Thus, since Cdc37 provides kinase specificity to the Hsp90 machinery, Cdc37^pY298^ may act as a platform for specific recruitment of modifying nRTKs into the kinase chaperone cycle of Hsp90.

The identification of multiple phosphorylation sites on both Hsp90 and its cochaperones suggests that the chaperone cycle may be regulated at the level of the machinery via a combinatorial phosphorylation pattern in a sequential and client class-specific manner. Support for this hypothesis comes from previous observations, where the same nRTK (Yes) was able to phosphorylate both Hsp90 and Cdc37, and from the current data showing a strong Cdc37^pY298^-SH2^Yes^ interaction (Fig. 6). The phosphomimetic mutant Y192E of Hsp90β or Y197E of Hsp90α prevents complex formation with Cdc37 and triggers the disassembly of the recruiting ternary complex irrespective of the phosphorylation state of Cdc37 (Fig. 4 and Supplementary Fig. 4)^20^. Therefore, phosphorylation at this position of Hsp90 must be tightly regulated and occur only after the client kinase has been loaded on the chaperone. In this respect, the ability of Y197 to become phosphorylated in the context of ternary complexes, shows a very strong correlation with the ability of Cdc37 to become phosphorylated at position Y298, since in the presence of a non-phosphorylatable Cdc37^Y298^ variant, Y197 (hsp90α) phosphorylation is greatly suppressed (Fig. 5). Mechanistically, the presence of a phosphotyrosine at position 298 and not the resulting C-Cdc37 domain unfolding is critical to promote phosphorylation of Hsp90 at Y197 (Fig. 5) and correlates with a higher effective concentration of Yes at the chaperone complex (Fig. 6e). *In cis* phosphorylation via SH2-mediated kinase recruitment, where an nRTK utilizes a phosphotyrosine on its own substrate as a docking site to further phosphorylate it on other accessible tyrosines is an efficient mechanism for substrate hyperphosphorylation through processive or non-processive mechanisms^57^. In the context of a macromolecular complex such as the ternary Hsp90-Cdc37-kinase complex, coupled phosphorylation occurs *in trans*, with the modifying kinase docked on Cdc37 while phosphorylating Hsp90. As not all Hsp90 tyrosine phosphorylation events are affected equally by Cdc37 phosphorylation (Fig. 5 and Supplementary Fig. 6), *in trans*-mediated coupled phosphorylation imprinted by Cdc37^Y298^ phosphorylation creates a unique phosphorylation pattern on Hsp90, where Y197 becomes phosphorylated at high levels, and is tailored for the progression of the kinase chaperone cycle. This highly regulated mechanism of Hsp90 phosphorylation is unique to tyrosine modification, as SH2 domains are only found in nRTKs. Therefore, it remains unknown how other phosphorylation events identified to impact the kinase chaperone cycle, such as the modification of S365 (Hsp90β) by CK2^39^, are regulated. Suppression of phosphorylation events by the action of phosphatases, combined with the sequential masking and unmasking of the modification sites during different steps of the chaperone cycle could provide alternative regulatory mechanisms.

## Methods

### Sample preparations and isotope labeling

Full-length human Cdc37 (Cdc37) and C-Cdc37 (a.a. 288–378) were cloned in a pDB.His.MBP vector and the catalytic domain of bRaf was cloned in a pDB.His.GST vector^14,40^. The plasmid encoding for residues 86–543 of human HOP was obtained from DNASU (#HsCD00530871). The SH2 domains of Yes, Src, Hck, and Lyn cloned in a pGEX vector that encodes for an N-terminal, GST tag, and a PreScission cleavage site were a gift from Bruce Mayer (Addgene plasmid # 46532, 46510, 46445, and 46452). The DNA encoding for the SH3 domain of Yes (amino acids 91–152) was synthesized for an *E. coli*-optimized codon usage (GeneArt) and cloned into a pDB.His.GST vector to produce a fusion protein with an N-terminal, His_6_-GST purification tag, and a TEV cleavage site, using the set of primers listed in Supplementary Table 1. The DNA encoding for full-length Hsp90αα1 and full-length Hsp90αβ1 cloned in a pET28 plasmid encoding for a His tag was a gift from the laboratories of Chad Dickey (USF Health) and Ernst Schonbrunn (Moffitt Cancer Center). Cdc37 and Hsp90 point mutants were generated using the QuikChange II XL Site-Directed Mutagenesis Kit (Agilent) and the set of primers listed in Supplementary Table 1.

Hsp90, Hop, Aha1, SH2, and SH3 constructs were transformed into BL21(DE3) (NEB). For the expression of Hsp90, Hop, and Aha1 constructs, cells were incubated at 37 °C until OD_600_ ~0.6 and then chilled for 10 min in a water/ice bath. Protein overexpression was induced by the addition of IPTG at a final concentration of 0.5 mM for 20 h at 18 ^o^C. For the expression of the SH2 and SH3 constructs, cells were incubated at 37 °C until OD_600_ ~0.6 and overexpression was induced by the addition of IPTG at a final concentration of 0.5 mM, for 5 h at 20 ^o^C. Protein labeling was performed using the same expression scheme. ^13^C/^15^N uniformly labeled proteins were produced in minimal media supplemented with ^15^NH_4_Cl and U-^13^C6 glucose. Methyl-group site-specific labeling of Val, Leu, Ile^δ^, Met, and Ala residues was performed in a perdeuterated background with the addition of 50 mg/L α-ketobutyric acid, 100 mg/L α-ketoisovaleric acid, 125 mg/L Met-[^2^H/^13^CH_3_], and 50 mg/L Ala-[^2^H/^13^CH_3_] to the media 40 min before induction^58,59^.

Cdc37 constructs, bRaf, Hop, and Aha1 were purified by two steps of Ni^2+^-affinity and size-exclusion chromatography^14,40^. Cdc37 was further purified over an anion exchange column. Cells overexpressing Hsp90 were resuspended in 20 mM Tris, pH = 8.0, 500 mM NaCl, 10 mM imidazole, 3 mM DTT, 1 mM PMSF, protease inhibitor cocktail, and 0.1 mg/ml lysozyme. Cells were disrupted by sonication and the lysate was clarified by centrifugation before loading to a Ni^2+^-affinity column. After extensive washing with lysis buffer, the Hsp90 was eluted in the same buffer containing 400 mM imidazole and loaded to a Superdex 200 26/600 in 50 mM Tris, pH = 8.0, 1 M NaCl, 0.5 mM EDTA, and 3 mM DTT. Finally, it was further purified through a 10-ml HiTrap Q Sepharose FF run with a 50 mM–1 M NaCl gradient in 25 mM Tris, pH = 7.5, 4 mM EDTA, and 3 mM DTT. Cells overexpressing the SH2 constructs were resuspended in 20 mM Tris, pH = 8.0, 150 mM NaCl, 0.5 mM EDTA and 5 mM b-mercaptoethanol, 1 mM PMSF, and 0.1 mg/ml lysozyme and lysed by sonication. The lysate was loaded on a GST fast-flow column and after extensive washing with lysis buffer, proteins were eluted with 40 mM glutathione. The fusion protein was cleaved with PreScission protease overnight at 4 °C and the GST tag was removed by running a second GST column. Finally, proteins were purified through a Superdex 75 column in 20 mM Tris, pH = 7.5, 100 mM NaCl, 0.5 mM EDTA, and 2 mM DTT. Cells overexpressing the SH3 construct of Yes were resuspended in 50 mM KPi, pH = 6.5, 150 mM NaCl, and 2 mM DTT and lysed by sonication. The same buffer was used throughout all purification steps. After centrifugation, the lysate was loaded on a GST column and the protein was eluted by 40 mM glutathione. The fusion protein was cleaved using TEV protease at 4 °C (overnight) and the SH3 domain was separated through a Ni^2+^ sepharose column (in the presence of 20 mM imidazole) and subsequently further purified through a Superdex 75.

The phosphotyrosine-modified decapeptide EVpYESLPEEL corresponding to the residues 296–305 of Cdc37 was synthesized by GL Biochem.

### NMR spectroscopy

All NMR spectra were acquired with Varian direct drive 600- and 800-MHz spectrometers equipped with a cryoprobe, processed using NMRpipe, and analyzed using Sparky (T. D. Goddard and D. G. Kneller, SPARKY 3, University of California, San Francisco, CS, USA). The methyl-group and backbone chemical shift assignment for wild-type C-Cdc37 was described previously^14,40^. Sequential ^1^H, ^13^C, and ^15^N backbone chemical shift assignment for C-Cdc37^Y298F^, C-Cdc37^Y298E^, and C-Cdc37 in the presence of 9 M urea, Yes^SH2^, and Yes^SH3^ was obtained by standard 3D triple-resonance experiments, acquired at 30 °C for C-Cdc37 and Yes^SH3^ constructs, and at 25 °C for Yes^SH2^. The backbone assignment of Yes^SH3^ at 30 °C was transferred to 5 °C by acquiring a set of five spectra at intermediate temperatures. The backbone assignment of C-Cdc37^Y298E^ was further facilitated by the use of selective amino acid labeling with ^15^N-Ala, -Gln, and -His, as well as tracing signals during the urea-unfolding transition of wild-type C-Cdc37. Methyl-group assignment of Cdc37^Y298E^ was obtained by tracing signals during the unfolding transition of wild-type C-Cdc37 together with a set of four valine mutants for M1, M316, M324, M337, and A329. The 3D HMQC–NOESY–HMQC spectrum of C-Cdc37^Y298E^ was acquired with a mixing time of 0.4 s at 30 ^o^C. The urea-unfolding transition of wild-type C-Cdc37 was performed by titrating two samples of equal concentration (0.4 mM) prepared in the absence or presence of 9 M urea into each other to obtain a series of urea concentrations in 0.5 M steps.

Protein dynamics for wild-type C-Cdc37 were described previously^40^. For C-Cdc37^Y298F^, ps–ns timescale motions were characterized by measuring {^1^H}–^15^N heteronuclear NOEs^60^, at 800 MHz, in the presence or absence of a 3 s presaturation period prior to the ^15^N excitation pulse and using recycle delays of 2 and 5 s, respectively, at 30 ^o^C. The data with and without NOE were acquired in a fid-interleaved fashion. *R*_*1*_ and *R*_*2*_^15^N relaxation rates were measured at 800 MHz, using standard pulse sequences^61^ with a recycle delay of 3 s, at 30 ^o^C. The delay periods in the series for *R*_1_ and *R*_2_ were set to 20 ( × 2), 50, 100 ( × 2), 200, 300, 400, 600, 800, 1000, 1200, 1500, and 2000 ms and to 10 (×2), 30, 50 ( × 2), 70 ( × 2), 90, 110, 130, 210, and 330 ms, respectively. Relaxation rate constants were determined by fitting Sparky-extracted peak heights to mono-exponential functions using relax^62^. Errors were determined by recording duplicate experiments for selected delay periods, noted by (×2) above. N–H vector motions were analyzed by the reduced spectral density-mapping approach and using scripts in relax.

The Yes^SH2^-phosphopeptide titration was performed at 25 °C by the addition of 1.2-molar equivalents of phosphopeptide at a concentration of 250 μM. Addition of higher excess of phosphopeptide did not cause any further changes in the ^15^N-HSQC spectrum of Yes^SH2^. The Yes^SH3^ titration was performed at 5 ^o^C, by the addition of 6.0 molar equivalents of unlabeled full-length Cdc37 to 15N-labeled Yes^SH3^, at 1.2 mM and 200 μM. Chemical shift perturbations are reported as H–NH-combined chemical shift changes, ∆*δ*, determined according to equation (1):

1 $$\Delta \delta = \sqrt {\Delta \delta _{\mathrm{H}}^2 + \left( {\frac{{\Delta \delta _{\mathrm{N}}}}{5}} \right)^2}$$

NMR spectra were acquired in 20 mM Tris, pH = 7.5, 100 mM NaCl, 0.5 mM EDTA, and 2 mM DTT prepared in either H_2_O or D_2_O (with Tris-d_6_), except for Yes^SH3^ that were acquired in 50 mM KPi, pH = 6.5, 150 mM NaCl, and 2 mM DTT.

The spectrum of phosphorylated Ile-labeled (^2^H/^13^C-CH_3_^δ^) Cdc37 was acquired by mixing 320 μL of Cdc37 at 25 μM, in 20 mM Tris-d_6_, pH = 7.5, 50 mM NaCl, 10 mM MgCl_2_, 0.2 mM EDTA, 3 mM DTT, 2.5 mM ATP and 1 × phosphatase inhibitors (Halt), and 7.5% D_2_O, with 15 μg of active human Yes kinase (EMD). The mixture was run through a desalting column (Zeba) equilibrated in the same buffer to remove residual glycerol coming from Yes stock, concentrated back to 320 μL, and put in a shigemi tube. The ^13^C-HMQC spectrum was acquired at 30 °C after incubation for 3 h at the same temperature. A reference spectrum of unmodified Cdc37 was acquired at the same temperature and the same buffer.

### Analytical size-exclusion chromatography

Size-exclusion chromatography was performed at 4 °C using a Biorad Enrich SEC 650 analytical column in 20 mM Tris, pH = 7.5, 100 mM NaCl, 0.5 mM EDTA, and 2 mM DTT. Analysis of ternary complexes was performed by mixing Hsp90 variants, Cdc37 variants, and bRaf at stoichiometric ratios of 2:1:1, while analysis of binary complexes by mixing Cdc37 variants and bRaf at stoichiometric ratios of 1:1. In both cases, the total volume was 200 μL and the mixtures were incubated for 15 min at 4 ^o^C. Protein molar concentrations (μM) ranged from 40:20:20 to 12:6:6 and 30:30 to 8:8 for ternary and binary complexes, respectively.

### Immunoprecipitation and pull down

Wild-type and mutant constructs of Hsp90 and Cdc37 have been previously described^20^. Briefly, to construct FLAG-tagged Hsp90α, BamHI and XhoI sites were engineered by PCR of the human Hsp90α cDNA (a kind gift from W. Houry, University of Toronto, Toronto, ON, Canada). The PCR product was subcloned into the BamHI/XhoI sites of the pcDNA3-FLAG vector (Invitrogen). FLAG-tagged wild-type Cdc37 in pcDNA3 vector was a kind gift from Dr. Y. Minami (University of Tokyo). To obtain HA-tagged Cdc37, we subcloned Cdc37 into HA-pcDNA3 plasmid following the manufacturer’s instructions (Invitrogen). Point mutations in both Hsp90 and Cdc37 were made using the QuikChange site-directed mutagenesis method following the manufacturer’s instructions (Stratagene). FLAG-tagged bRaf plasmid was purchased from Biomyx (pMEV-HA 2×). Glucocorticoid receptor plasmid (untagged) was kindly provided by Dr. M. Cox (University of Texas at El Paso). GFP-tagged androgen receptor (AR) plasmid was a kind gift of Dr. Lisa Butler (University of Adelaide). All antibodies and other reagents were commercially obtained. HEK-293 cells were purchased from ATCC, and were maintained in culture and transfected as previously described (Xu et al., 2012). Briefly, cells were grown in DMEM tissue culture medium containing 10% fetal bovine serum, and cells were transfected with Lipofectamine 2000 following the manufacturer’s instructions. Proteins were immunoprecipitated and subjected to SDS-PAGE and western blotting as described in figure legends. Briefly, 24 h after transfection, cells were washed with PBS and lysed in a Hepes buffer containing 10 mM Na_2_MoO_4_, 30 mM NaF, 2 mM β-glycerol phosphate, 2 mM sodium vanadate, 100 µM bpv(phen), and Complete protease inhibitors (Roche Applied Science, Indianapolis, IN). After immunoprecipitation (see individual figure legends for antibodies used for immunoprecipitation), proteins were boiled in sample-loading buffer, resolved by SDS-PAGE, and transferred onto PVDF membrane. Membranes were probed with indicated antibodies (see figure legends). Antibody sources/clone #’s are as follows: GR (glucocorticoid receptor) monoclonal antibody is from Santa Cruz Biotechnology (cat # sc-393232, 1:5000); GFP monoclonal antibody is from Cell Signaling (cat #2956, 1:1000); GFP-trap beads are from Chromotek (GFP-Trap A, cat # gta-20); anti-FLAG monoclonal antibody (clone M2) is from Sigma (cat # F3165, 1:2000); anti-FLAG resin is from Sigma (M2 anti-FLAG antibody-linked resin); Hsc/Hsp70 antibodies are from Santa Cruz (cat # sc-1059, 1:1000, sc-1060, 1:1000); Aha1 antibody is from Rockland (cat # 600-401-974); p23 antibody is from Assay Designs (ADI-SPA-610, 1:1000); HOP antibody is from Cell Signaling (cat # 4464, 1:1000); FKBP59 antibody is from StressMarq (SMC-139, 1:1000); Cdk4 antibody is from Santa Cruz (sc-601, 1:1000); HA antibody is from Roche diagnostics (rat anti-HA, clone 3F10, 1:1000); anti-c-Myc Agarose Affinity Gel antibody from Sigma (cat # A7470); and penta·His Antibody, BSA-free, is from Qiagen (cat # 34660, 1:5000). Uncropped scans of the blots and gels are provided in Supplementary Fig. 5.

### Fluorescence spectroscopy

Trp fluorescence for wild-type, Y298F, and Y298E C-Cdc37 as a function of urea concentration was carried out on a ISS PC1 single-photon-counting fluorimeter at protein concentrations of 5 μM. Spectra were acquired with a 1-nm step interval between 310 and 450 nm and with an excitation wavelength of 395 nm. Signal intensity at each interval is an average of eight iterations.

### Isothermal titration calorimetry

Titrations were carried out on a PEAQ-ITC calorimeter (Malvern Scientific) at 20 °C for titrations of bRaf into Cdc37 variants and at 25 °C for phosphopeptide titrations into SH2 domains. Titrations of cochaperones to Hsp90β and Hsp90α constructs were performed at 25 and 36 ^o^C, respectively. Proteins were buffer exchanged into 20 mM Tris, 100 mM NaCl, 0.5 mM EDTA, and 1 mM tris(2-carboxyethyl)phosphine and degassed. For Cdc37-bRaf titrations, the 200-μL sample cell was filled with Cdc37 at a concentration of ~3–8 μM protein and the 40-μl injection syringe was filled with bRaf at 35–90 μM. For Hsp90-cochaperone titrations, the cell was filled with Hsp90 at a concentration of ~20–30 μM and the syringe with the cochaperone at a concentration of ~300–400 μM. For the SH2-phosphopeptide titrations, the cell was filled with the SH2 domain at a concentration of 50–60 μM and the injection syringe was filled with phosphopeptide at a concentration of 600–750 μM. All titrations included an initial 0.2-μL injection and were carried out by 10–12 injections, with a 4-min time interval between each injection. The data were processed with Origin 7.0 (OriginLab Corporation) with the point of the initial injection excluded. For all experiments, the reported error bars in the *K*_d_ values correspond to the errors resulted in fitting of the data into a single binding site model.

### CD experiments

Thermal denaturation of wild-type, Y298F, and Y298E C-Cdc37 for the extraction-melting temperatures (T_m_) was performed by monitoring molar ellipticity at 222 nm, using an AVIV (215) Circular Dichroism Spectrometer. Full spectra between 195 and 250 nm were acquired at 15, 22, and 30 ^o^C. Protein samples were at 5 μM in 20 mM Tris, pH = 7.5, 100 mM NaCl, 0.5 mM EDTA, and 2 mM DTT. Signal intensity at each wavelength is reported as an average of triplicate measurements, each of which was obtained using a signal-averaging time of 1 s. The RMSDs ranged between 0.1 and 5.0 across the full spectrum.

### In vitro phosphorylation and detection

Proteins were exchanged in 20 mM Tris, pH = 7.5, 50 mM NaCl, 0.2 mM EDTA, and 2.5 mM DTT and mixed to achieve final protein molar concentrations (μM) of 24:12:12 and 12:12 for ternary Hsp90-Cdc37-bRaf and binary Cdc37-bRaf complexes, respectively, at a final volume of 12 μL. The same concentrations were used for free, Hsp90, and Cdc37 variants. Mixtures were incubated at 4 °C for 15 min and the reactions were initiated by the addition of phosphatase inhibitor cocktail, 0.91 μg of recombinant full-length Yes (EMD), and ATP (0.5 mM final concentration), at 22 ^o^C. Phosphorylation was tested after 2 or 8 h, but final analysis is provided only for the 2-h interval, as the levels either decrease or remain constant at longer incubation times for different tyrosine residues. For analysis by mass spectrometry, 6 μl of each reaction was run on SDS-PAGE. Coomassie-stained gel pieces corresponding to Hsp90 and Cdc37 were excised from the gel, minced and destained before being reduced with dithiothreitol (DTT) and alkylated with iodoacetamide (IAA), and finally digested with trypsin/Lys-C overnight at 37 ˚C. Peptides were extracted using 50/50 acetonitrile (ACN)/H_2_O/0.1% formic acid, and dried in a vacuum concentrator (Labconco). Peptides were resuspended in 98% H_2_O/2% ACN/0.1% formic acid for LC–MS/MS analysis and separated using a 75 µm x 50 cm C18 reversed-phase-HPLC column (Thermo Fisher Scientific) on an Ultimate 3000 UHPLC (Thermo Fisher Scientific) with a 60-min gradient (4–40% ACN with 0.1% formic acid). Analysis was performed on a hybrid quadrupole-Orbitrap instrument (Q Exactive Plus, Thermo Fisher Scientific). Full MS survey scans were acquired at 70,000 resolution. The top 10 most abundant ions were selected for MS/MS analysis. Raw data files were processed in MaxQuant (v.1.5.8.3 www.maxquant.org). Spectra were identified using Andromeda, the MaxQuant peptide identification algorithm, and searched against the UniprotKB human protein sequence database, with constant modification of cysteine by carbamidomethylation and the variable modification, methionine oxidation, and phosphorylation of serine, threonine, and tyrosine. Trypsin was specified as the protease, with a maximum of two possible missed cleavages. Additionally, the database search specified mass tolerance of 20 ppm (first search) and 4.5 ppm (recalibrated, second search) for precursor ions, and 20 ppm for fragment ions. Proteins were identified using the filtering criteria of 1% protein and peptide false-discovery rate.

In total, two phosphorylation sites were detected for Cdc37 (Y298 and Y331) and nine phosphorylation sites were detected for Hsp90 (Y61, Y160, Y197, Y284, Y309, Y438, Y492, Y604, and Y667). The results are displayed as normalized ratios of phosphorylated over non-phosphorylated peptides and errors were calculated by quantifying phosphorylation in a set of two different reactions. Ratios are provided only for those sites that the corresponding peptide was identified in both reactions (Y197, Y61, Y160, Y438, Y492, and Y604). For analysis by ProQ-Diamond staining, the fraction of the in vitro phosphorylation reaction that was run on SDS-PAGE contained ~3.0 μg of Hsp90. Staining and destaining were performed by standard protocols.

### Molecular dynamics simulations

Initial structures of the helical core (residues 290–343) of the C-terminal domain of Cdc37 were taken from the protein data bank (PDB ID 2N5X). Systems were built using CHARMM^63^, but run with OpenMM^64^ using the CHARMM force field^65^. Three different systems were modeled, the unphosphorylated and Y298 phosphorylated wild types, and the Y298E mutant. Systems were solvated in TIP3P^66^ water boxes, with at least 20 Å of solvent beyond the protein in all directions. A nonbonded cutoff of 12.0 Å was used. Long-range electrostatic interactions were handled using the particle mesh Ewald method^67^. Heating occurred with restraints of 5 kcal/(mol Å) on the backbone atoms from 150 K to either 300 K or 310 K, in intervals of 10 K and 20 ps. Backbone restraints were then gradually released to 2.5, 1.0, 0.5, and 0.1 kcal/(mol Å) over 10 ns of further simulation, followed by production runs. The NPT ensemble was used with Langevin Dynamics and a Monte Carlo Barostat^68^. Visualization was performed with VMD^69^, and AmberTools was used for the other analyses^70^.

### Data availability

The mass spectrometry proteomics data have been deposited to the ProteomeXchange Consortium via the PRIDE partner repository with the dataset identifier PXD008375. Chemical shifts of C-Cdc37^Y298E^, C-Cdc37^Y298F^, and C-Cdc37 in the presence of 9 M urea, free Yes^SH2^, Yes^SH2^ in complex with pYESL, and Yes^SH3^ are deposited at BMRB, under accession numbers 27322, 27323, 27324, 27325, 27326, and 27327, respectively. All other data that support the findings of this study are available from the corresponding author upon reasonable request.

## Electronic supplementary material

Supplementary Information
