# Supplementary material for: Phosphorylation induced cochaperone unfolding promotes kinase recruitment and client class-specific Hsp90 phosphorylation
Source: Nat Commun. 2018 Jan 17;9:265. doi: 10.1038/s41467-017-02711-w (PMC5772613; doi:10.1038/s41467-017-02711-w)
Supplement: Supplementary file 1 — Supplementary Information [file 41467_2017_2711_MOESM1_ESM.pdf]

SUPPLEMENTARY FIGURE 1

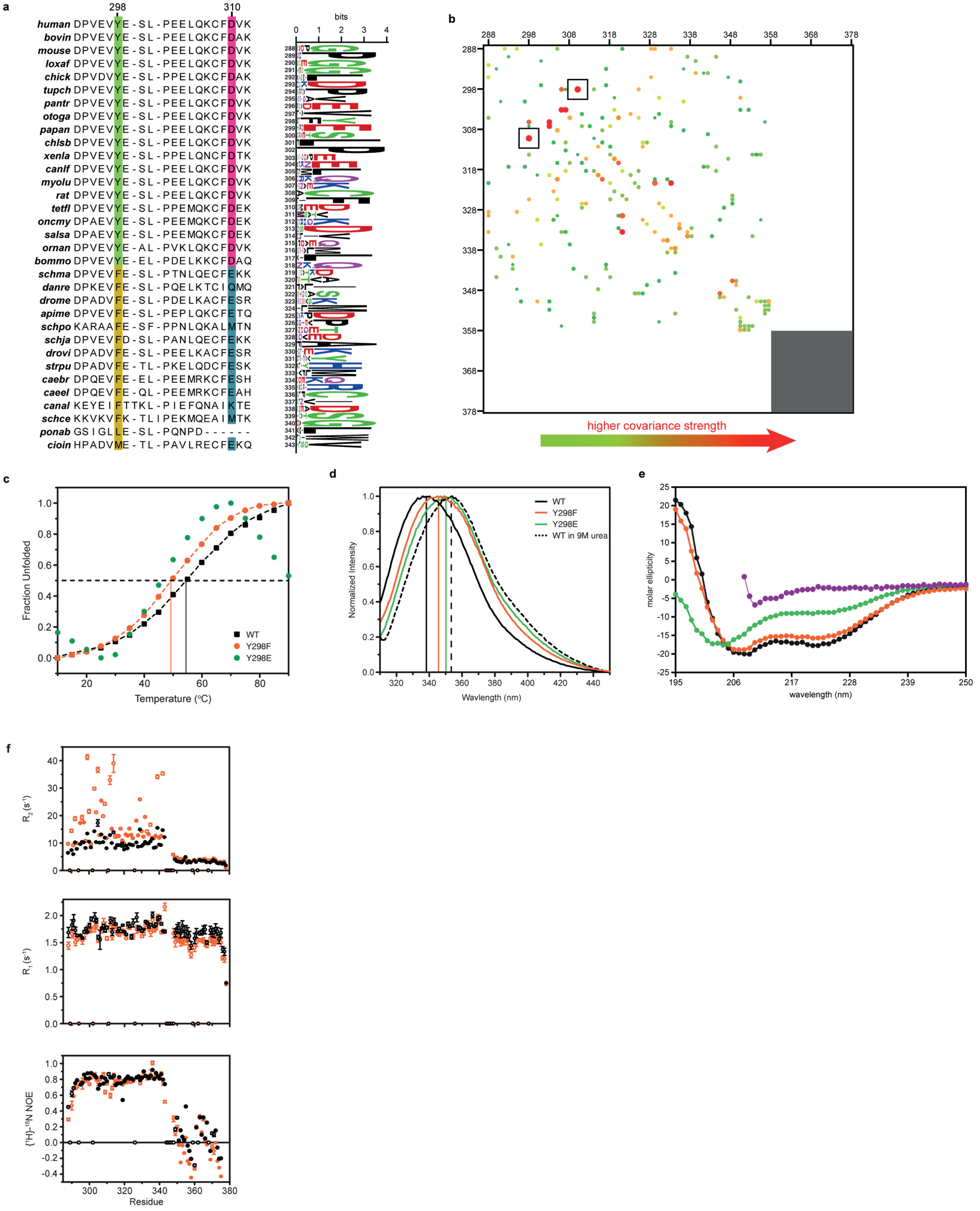

Supplementary Figure 1 Analysis of the sequence and biophysical properties of C-Cdc37 (**a**) A segment of Cdc37's multiple sequence alignment generated using JalView (left)[1], covering helices α_1 and α_2 of C-Cdc37, which encompass Y298 and D310, respectively, together with a sequence logo generated using WebLogo[2] corresponding to the structured region (288-343) of the domain (right). (**b**) C-Cdc37 residue pairs sorted by coevolution strength, where the strength in covariance increases from green to red and with increasing radius. Only covarying residues with sequence separation greater than three amino acids were considered. The last 20 amino acids of Cdc37 were excluded from the analysis as the multiple sequence alignment for this segment contained > 75% gaps. The Y298-D310 pair (boxed) shows the strongest covariation within C-Cdc37. The coevolution matrix was generated using gremlin[3]. (**c**) Thermal denaturation curves of wild-type (black), Y298F (orange) and Y298E (green) C-Cdc37 obtained by measuring molar ellipticity as a function of temperature. (**d**) Fluorescence emission spectra of wild-type (black), Y298F (orange) and Y298E (green) C-Cdc37 acquired in native buffer. For comparison, the spectrum of wild-type C-Cdc37 was also acquired under denaturing conditions, in the presence of 9M urea. The frequency of maximum emission of each spectrum is marked on the x-axis. C-Cdc37 contains a single tryptophan at position 342. (**e**) CD spectra of wild-type (black), Y298F (orange) and Y298E (green) C-Cdc37 acquired in native buffer at 15 ^{o}C, and of wild-type C-Cdc37 acquired in the presence of 9M urea (purple). (**f**) ^{15}N relaxation rates R_2, R_1 and ^{1}H-^{15}N NOE of wild-type (black) and Y298F C-Cdc37 (orange).

SUPPLEMENTARY FIGURE 2

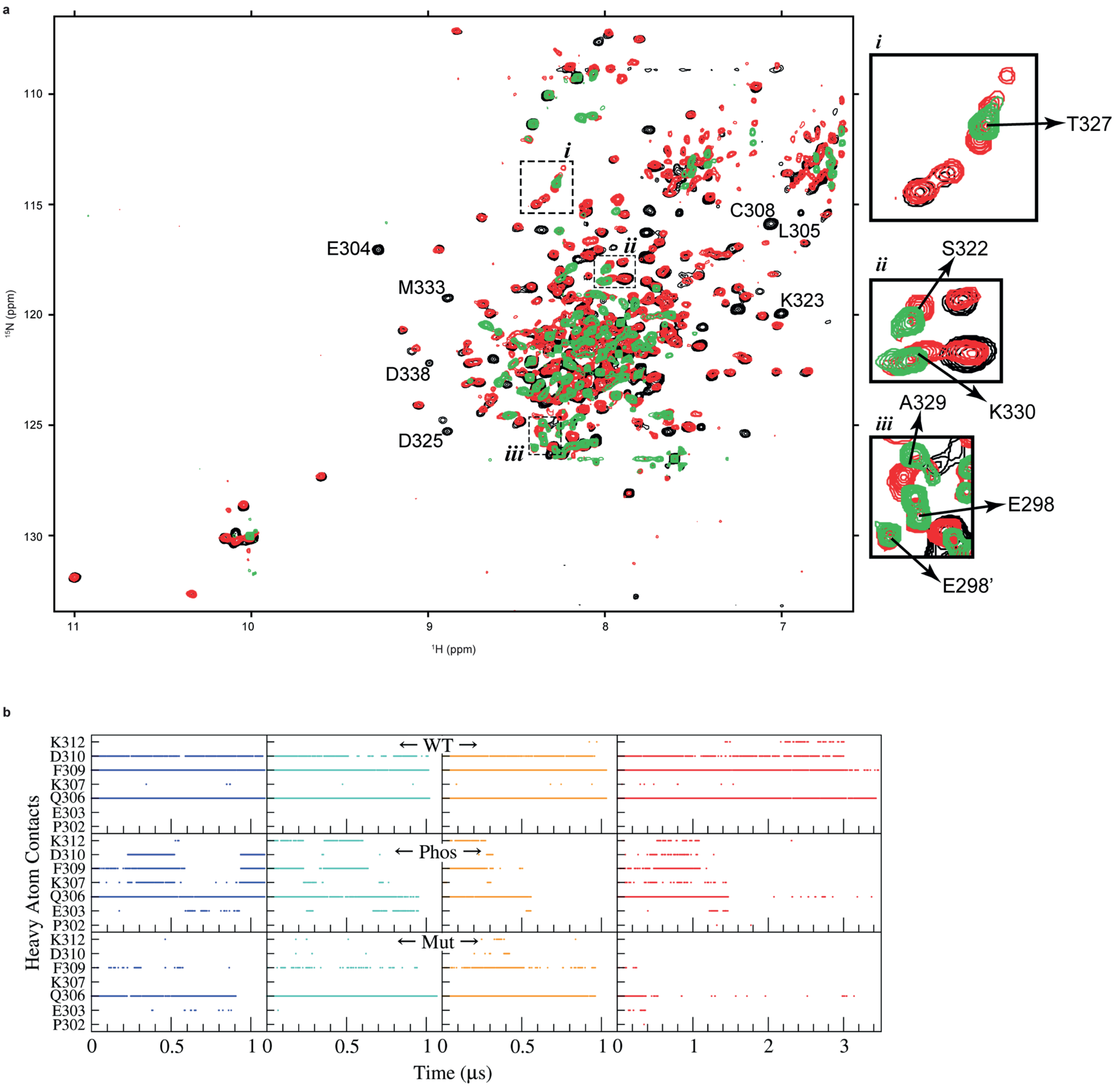

Supplementary Figure 2 Phosphorylation-induced unfolding of C-Cdc37. (**a**) Overlay of the ^{15}N-HSQC spectra of full-length wild-type Cdc37 (black), full-length Cdc37^{Y298E} (red) and C-Cdc37^{Y298E} (green). The assignment of a set of dispersed C-Cdc37 signals is shown to highlight that the loss of dispersion observed in the spectrum of C-Cdc37^{Y298E} (Figure 2A) is also observed in the spectrum of full-length Cdc37^{Y298E}. The expanded regions depicted on the right show that there is a very good correspondence between the position of new peaks of full-length Cdc37^{Y298E} and C-Cdc37^{Y298E}, indicating that the phosphomimetic mutation causes the same conformational rearrangement to full-length Cdc37 (partial unfolding) as to isolated C-Cdc37. (**b**) Heavy atom contacts within 4 Å between residue 298 and helix α_2 as derived from the molecular dynamics simulations performed on wild-type (top), phosphorylated (Y298) (middle) or the Y298E phosphomimetic mutant of C-Cdc37 (bottom).

SUPPLEMENTARY FIGURE 3

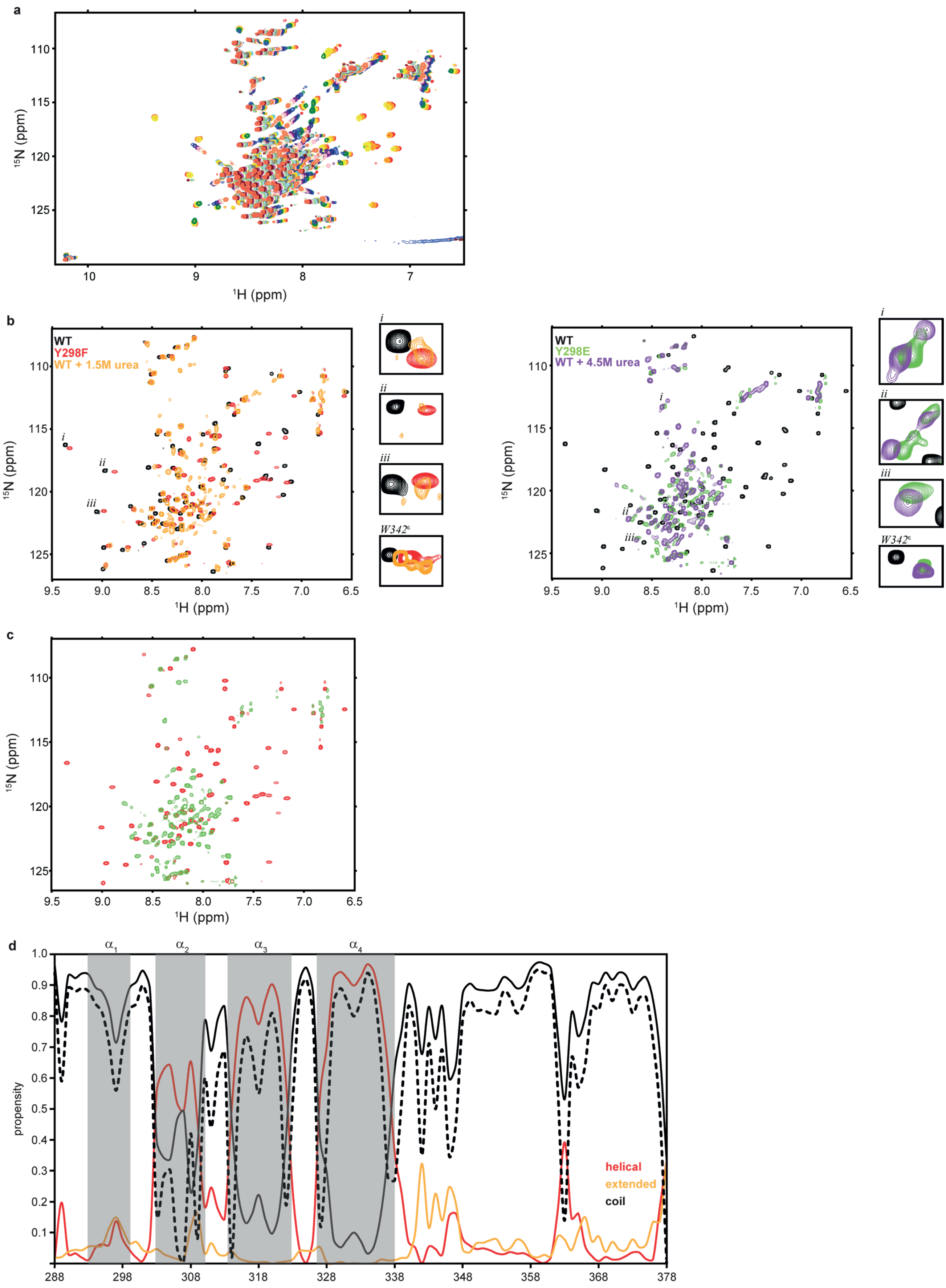

Supplementary Figure 3 Characterization of the partially unfolded state induced by the phosphomimetic mutation (**a**) The unfolding transition of C-Cdc37 monitored using ^{15}N-HSQCs recorded in the presence of different urea concentrations ranging from 0 to 9 M at 0.5 M steps. (**b**) Comparison of the ^{15}N-HSQC spectra of wild-type C-Cdc37 under native conditions (black), Y298F under native conditions (red) and wild-type C-Cdc37 in the presence of 1.5 M urea is shown on the left. The same comparison is shown on the right, but for Y298E (green) and wild-type C-Cdc37 in the presence of 4.5 M urea (purple). In the expansions of selected regions, the spectra acquired under non-native conditions are shown at a lower threshold. (**c**) A comparison of the ^{15}N-HSQC spectra of Y298F (red) and Y298E (green) C-Cdc37. (**d**) Secondary structure propensity of Y298E derived by analyzing the backbone C^a, C^b, N, C' and H^N chemical shifts using Talos-N[4]. Helical, extended and coil conformations are shown as continuous lines in red, yellow and black respectively. The confidence of prediction is shown as broken black line.

SUPPLEMENTARY FIGURE 4

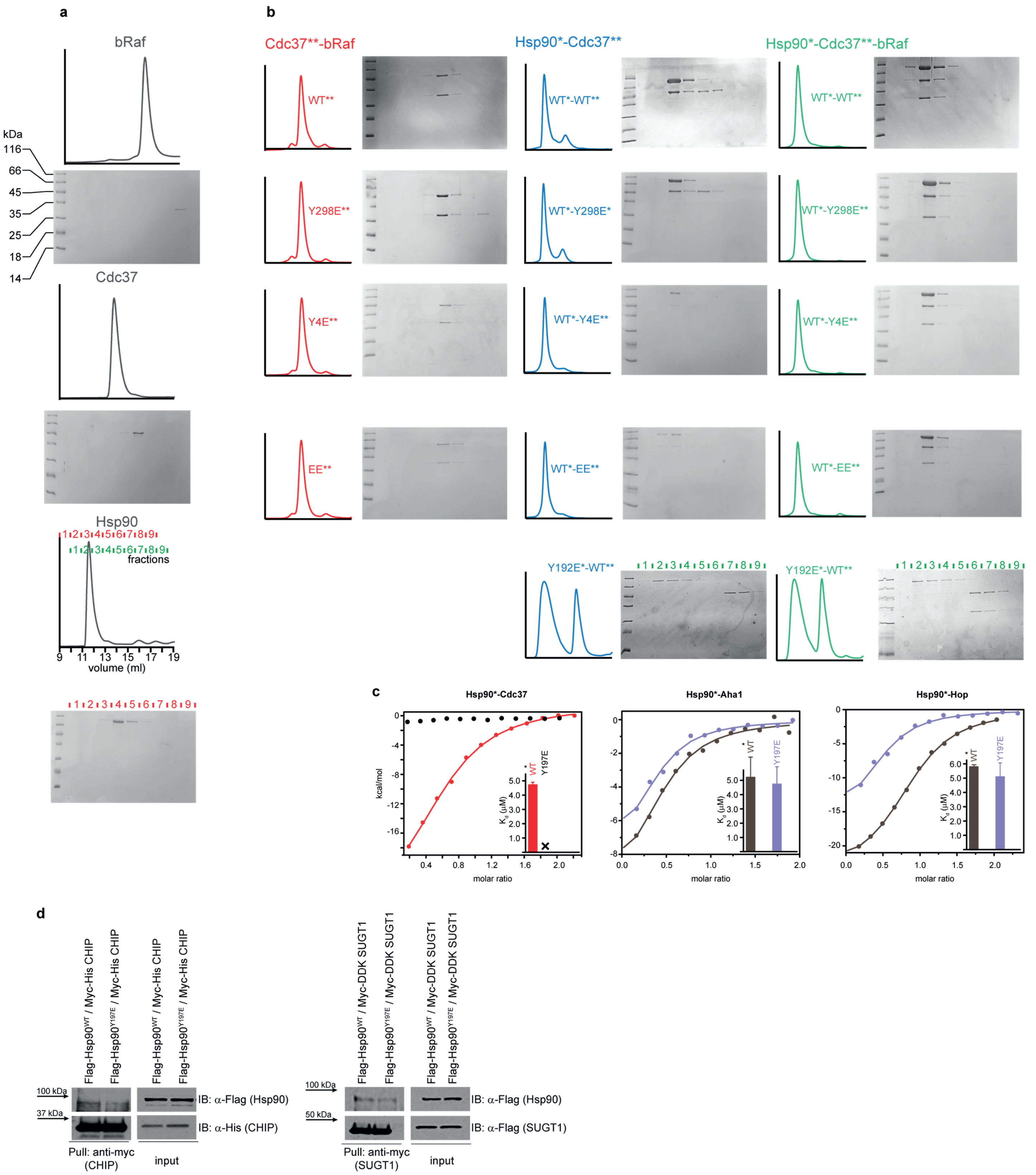

Supplementary Figure 4 The effect of phosphomimetic-related mutation on the assembly of Hsp90 and Cdc37 binary and ternary complexes with other cochaperones and the client kinase bRaf. (**a**) Size exclusion chromatography traces and the corresponding gels of free bRaf, Cdc37 and Hsp90. The elution volume and the fractions analyzed by SDS-PAGE is shown for reference for Hsp90. (**b**) Size exclusion chromatography traces

and the corresponding SDS-PAGE images for binary complexes of bRaf with Cdc37 variants (red), binary complexes of Hsp90 and Cdc37 variants (blue) and ternary complexes of bRaf with Cdc37 and Hsp90 variants (green). All gels were loaded according to the fraction scheme shown for Hsp90 in (marked in red in (**a**)), with the exception of the Hsp90^{Y192E} gels that were loaded with the fraction scheme marked in green in (**a**). The same molecular weight marker as the one shown in the top panel of (**a**) was loaded on all gels. (**c**) The interaction of Hsp90α and its phosphomimetic mutant Y197E with the cochaperones Cdc37, Aha1 and Hop, followed by ITC. The corresponding K_ds are shown in the inserts. (**d**) HEK-293 cells were transfected with indicated plasmids and lysed in 20 mM Hepes pH 7.3, 100 mM NaCl, 1 mM $MgCl_2$, 0.1% NP-40, 10 mM sodium molybdate, plus protease and phosphatase inhibitors. Proteins were precipitated with indicated antibody-conjugated resin for 1h at 4 ^{o}C with rotation. Bead pellets were washed three times with lysis buffer before analysis via SDS-PAGE and western blot.

SUPPLEMENTARY FIGURE 5

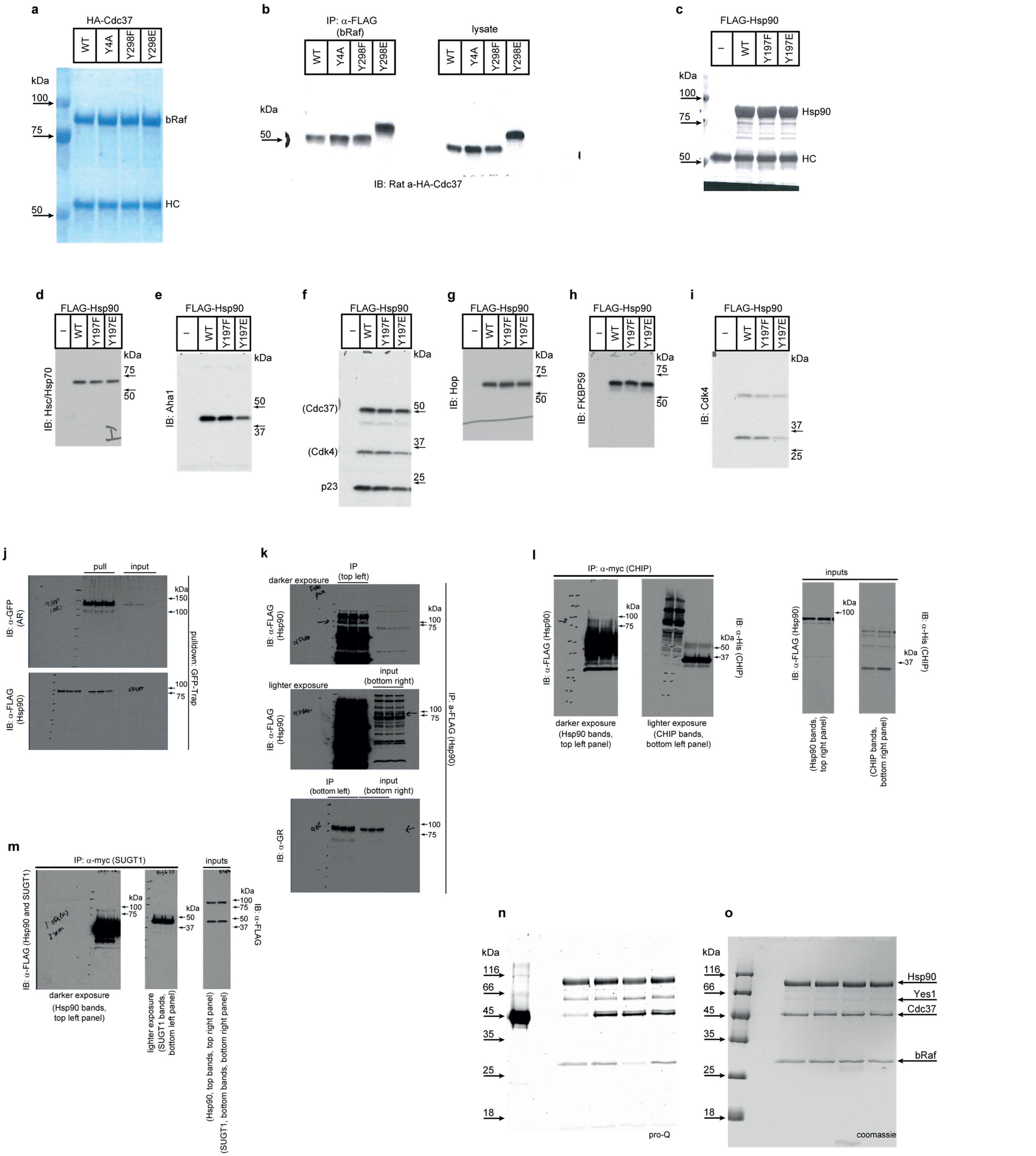

Supplementary Figure 5 Scans of uncropped gels shown in the main text and in the Supplemental information. Fig. 4b (**a-b**), Fig. 4c (**c-i**), Fig. 4d (**j-k**), Supplementary Fig. 4 (**l-m**) and Fig. 6 (**n-o**).

SUPPLEMENTARY FIGURE 6

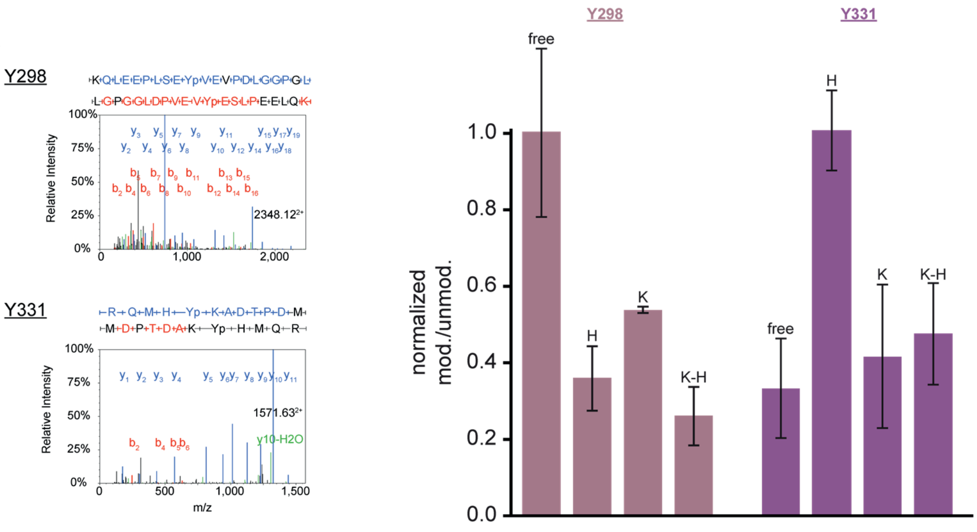

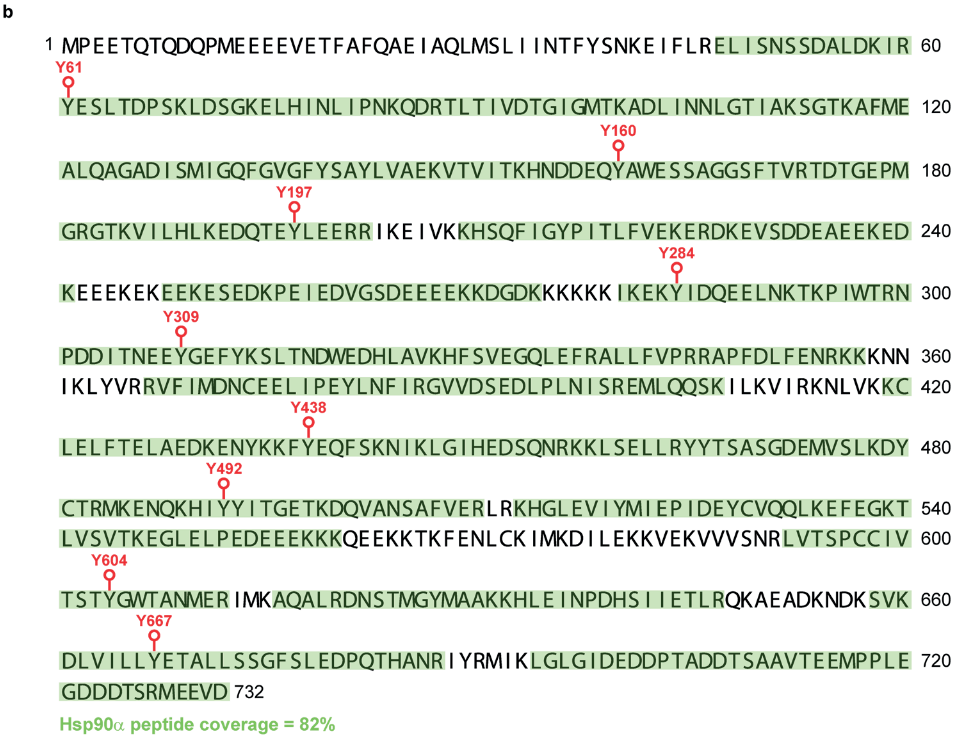

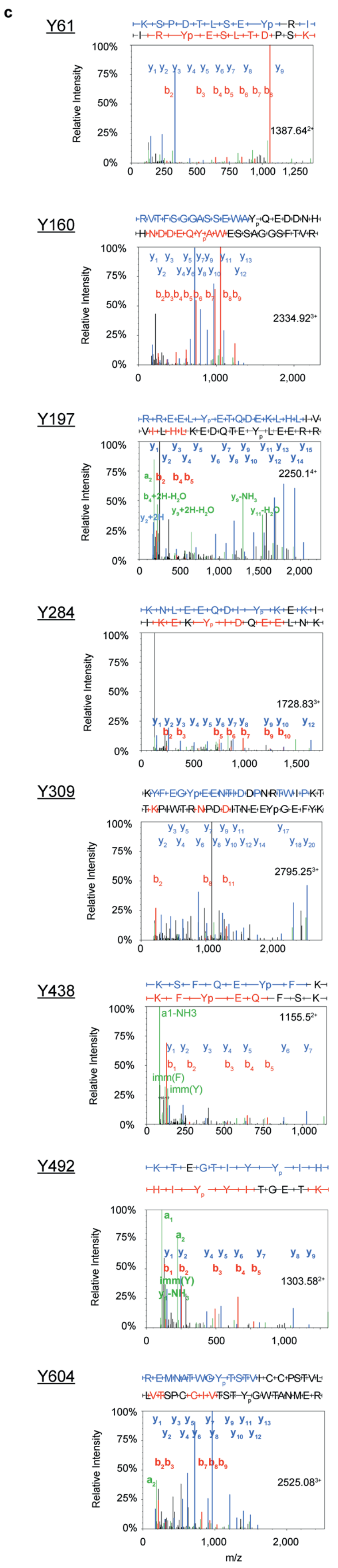

Supplementary Figure 6 Cdc37 and Hsp90 phosphorylation sites identified in this study by mass spectrometry at different liganded states. (**a**) MS/MS spectra of Cdc37 phosphopetides for Y298 and Y331 (left), and ratios of modified over unmodified peptides for Y298 and Y331 of Cdc37. (**b**) The sequence of Hsp90 (alpha isoform)

highlighting in green the peptide coverage obtained in the current study and in red the tyrosine residues identified in the phosphorylated form. (**c**) MS/MS spectra of Hsp90 phosphopetides identified in the current study.

SUPPLEMENTARY FIGURE 7

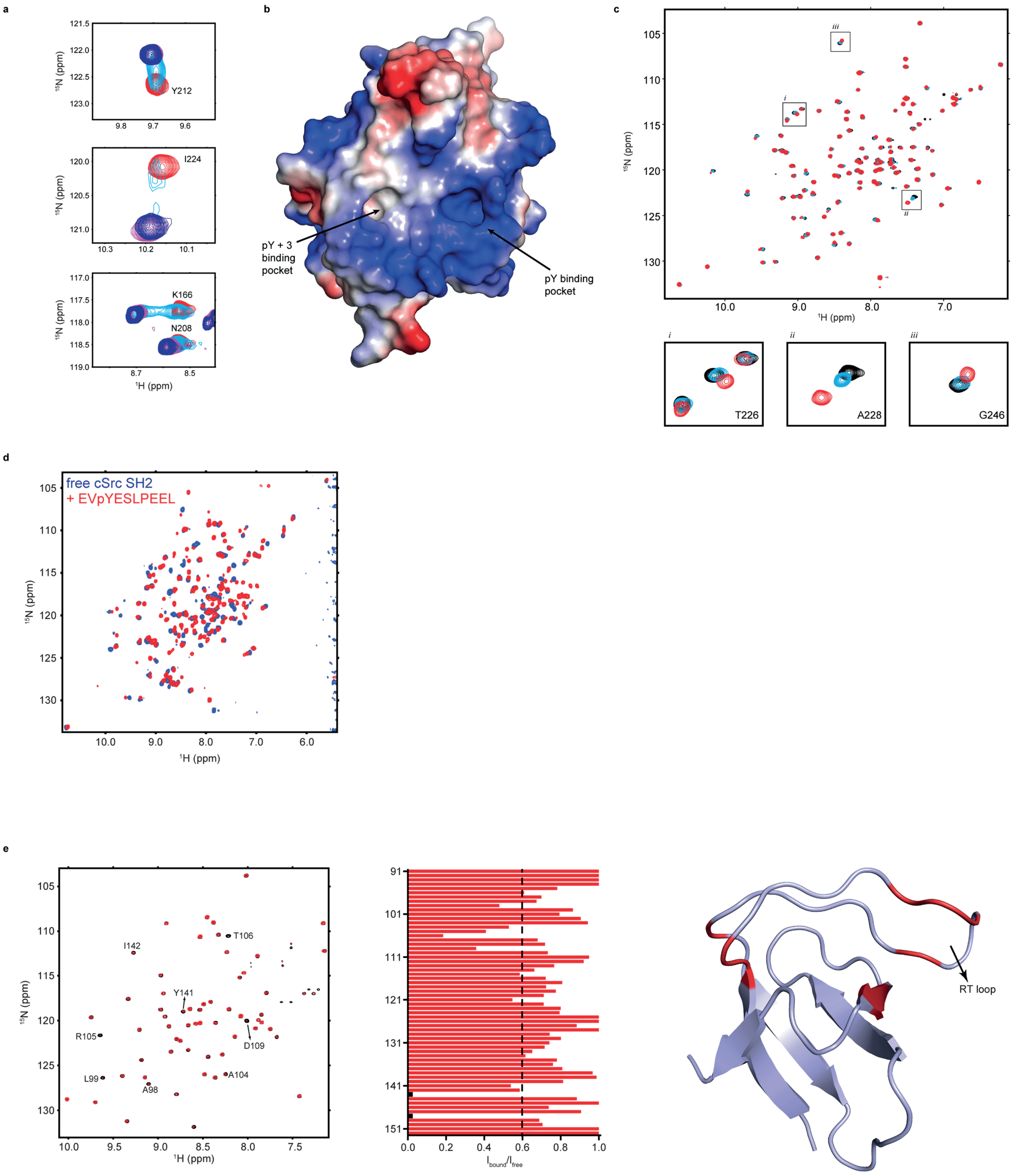

Supplementary Figure 7 Interaction of SH2 and SH3 regulatory domains of nRTKs with Cdc37. (**a**) Expanded regions of the ^{15}N-HSQC of Yes^{SH2} (red) in the presence of 0.5 (cyan), 1.0 (magenta) and 1.2 (blue) equivalents pYESL. (**b**) The electrostatic potential of SH2^{Yes} mapped on its structure (5MTJ). The orientation of the domain

is the same as for Fig. 6d. (**c**) Overlay of the ^{15}N-HSQC spectra of free Yes^{SH2} (black) and in the presence of two equivalents wild-type C-Cdc37 (cyan) or C-$Cdc37^{Y298E}$ (red). The signals of T226, A228 and G246 which show the largest chemical shift are found in the vicinity of the pY+3 binding pocket. (**d**) Overlay of the ^{15}N-HSQC of Src^{SH2} in the absence (blue) and presence (red) of one equivalent pYESL. (**e**) Left: overlay of the ^{15}N-HSQC of free Yes^{SH3} (black) and the presence of five molar equivalents of full-length Cdc37 (red), acquired at 5 ^{o}C. Signal assignment is shown for those residues that experience a significant drop in intensity in the presence of Cdc37. Middle: plot of intensities of $SH3^{Yes}$ signals in the presence over the corresponding intensities in the absence of full-length Cdc37. The dotted line marks a drop equal to one standard deviation higher than the average drop. Right: mapping of the residues affected by Cdc37 on the crystal structure of $SH3^{Yes}$.

Supplementary Table1: List of primers used in the present study

Mutant	PRIMER
F-yes-SH3	GGAATTCCATATGGGTGGTGTTACCATTTTTGTTG
R-yes-SH3	CGCGGATCCTTATTAGCTATCTGCCGGTGCAAC
F-Cdc37-Y298E	GCCGCCGGGGCCAACCCGCTTCTTGCG
R-Cdc37-Y298E	CGCAAGAAGCGGGTTGGCCCCGGCGGC
F-Cdc37-Y298F	GGGAGGGACTCGAAGACCTCGACGG
R-Cdc37-Y298F	CCGTCGAGGTCTTCGAGTCCCTCCC
F-Cdc37-D310A	CATCTGCACGTCCTTCACCGCGAAGCACTTCTGGAGTTC
R-Cdc37-D310A	GAACTCCAGAAGTGCTTCGCGGTGAAGGACGTGCAGATG
F-Cdc37-Y4E	GTGGTCCCACACGCTTTCGTCCACCATATGGCC
R-Cdc37-Y4E	GGCCATATGGTGGACGAAAGCGTGTGGGACCAC
F-Cdc37-Y4F	GGTCCCACACGCTGAAGTCCACCATATGG
R-Cdc37-Y4F	CCATATGGTTGGACTTCAGCGTGTGGGACC
F-Hsp90b-Y192E	TGACCCGCCTCTCTTCTAGTTCCCTTGTCTGATCTTCTTTA
R-Hsp90b-Y192E	TAAAGAAGATCAGACAGAGGAACTAGAAGAGAGGCGGGTCA
F-Hsp90a-Y197E	CTTTTATTCTTCGTTCCTCCAATTCCTCAGTTTGGTCTTCTTTCAGG
R-Hsp90a-Y197E	CCTGAAAGAAGACCAAACTGAGGAATTGGAGGAACGAAGAATAAA GG
F-Hsp90a-Y197F	TTATTCTTCGTTCCTCCAAGAACTCAGTTTGGTCTTCTTTC
R-Hsp90a-Y197F	GAAAGAAGACCAAACTGAGTTCTTGGAGGAACGAAGAATAA

Supplementary References:

1. Waterhouse, A.M., Procter, J.B., Martin, D.M., Clamp, M. & Barton, G.J. Jalview Version 2--a multiple sequence alignment editor and analysis workbench. *Bioinformatics* **25**, 1189-91 (2009).
2. Crooks, G.E., Hon, G., Chandonia, J.M. & Brenner, S.E. WebLogo: a sequence logo generator. *Genome Res* **14**, 1188-90 (2004).
3. Ovchinnikov, S., Kamisetty, H. & Baker, D. Robust and accurate prediction of residue-residue interactions across protein interfaces using evolutionary information. *Elife* **3**, e02030 (2014).
4. Shen, Y. & Bax, A. Protein backbone and sidechain torsion angles predicted from NMR chemical shifts using artificial neural networks. *J Biomol NMR* **56**, 227-41 (2013).
